# Spinal neural tube closure depends on regulation of surface ectoderm identity and biomechanics by Grhl2

**DOI:** 10.1038/s41467-019-10164-6

**Published:** 2019-06-06

**Authors:** Evanthia Nikolopoulou, Caroline S. Hirst, Gabriel Galea, Christina Venturini, Dale Moulding, Abigail R. Marshall, Ana Rolo, Sandra C. P. De Castro, Andrew J. Copp, Nicholas D. E. Greene

**Affiliations:** 1 Developmental Biology and Cancer Programme, UCL Great Ormond Street Institute of Child Health, University College London, 30 Guilford Street, London, WC1N 1EH United Kingdom; 2 UCL Infection and Immunity Division, UCL Pathogen Genomic Unit, UCL Cruciform Building, Gower Street, London, WC1E 6BT United Kingdom; 3 Present Address: Horizon Discovery, 8100 Cambridge Research Park, Cambridge, CB25 9TL United Kingdom; 4 Present Address: Instituto de Medicina Molecular, Faculdade de Medicina da Universidade de Lisboa, Avenida Professor Egas Moniz, 1649-028 Lisboa, Portugal

**Keywords:** Disease model, Embryogenesis, Morphogenesis, Organogenesis

## Abstract

Lack or excess expression of the surface ectoderm-expressed transcription factor Grainyhead-like2 (Grhl2), each prevent spinal neural tube closure. Here we investigate the causative mechanisms and find reciprocal dysregulation of epithelial genes, cell junction components and actomyosin properties in Grhl2 null and over-expressing embryos. Grhl2 null surface ectoderm shows a shift from epithelial to neuroepithelial identity (with ectopic expression of N-cadherin and Sox2), actomyosin disorganisation, cell shape changes and diminished resistance to neural fold recoil upon ablation of the closure point. In contrast, excessive abundance of Grhl2 generates a super-epithelial surface ectoderm, in which up-regulation of cell-cell junction proteins is associated with an actomyosin-dependent increase in local mechanical stress. This is compatible with apposition of the neural folds but not with progression of closure, unless myosin activity is inhibited. Overall, our findings suggest that Grhl2 plays a crucial role in regulating biomechanical properties of the surface ectoderm that are essential for spinal neurulation.

## Abstract (web summary)

Loss or over-expression of Grainyhead-like transcription factors (Grhl) prevents closure of the neural tube but the mechanism underlying this is unclear. Here, the authors show that Grhl2 regulates murine posterior-neuropore closure via changes in the identity and biomechanics of the non-neural, surface ectoderm cells.

## Introduction

The vertebrate neural tube, precursor of the brain and spinal cord, forms by progressive adhesion and tissue fusion of paired neural folds along the rostro-caudal extent of the embryonic body axis. The apposing neural folds are two-layered structures comprising neuroepithelium overlaid by non-neural, surface ectoderm. Much attention has focussed on the role of the neuroepithelium in the closure process. However, several lines of evidence suggest that the surface ectoderm, despite being only of single cell thickness, also plays a crucial role in neural tube closure. It is a source of signalling molecules (e.g., BMPs) that regulate neural fold bending in the lower spine^1^. Surface ectoderm cells also directly contribute to propagation of closure. In both the mid-hindbrain and the spinal region, it is surface ectoderm cells at the border with the neuroepithelium that mediate initial contact of the neural folds^2,3^. Hence, abnormalities in either the neuroepithelium or surface ectoderm may prevent closure, resulting in neural tube defects (NTDs), such as spina bifida^4–6^.

Among surface ectoderm-expressed genes in mice, closure of the neural folds along almost the entire length of the body axis depends on *Grhl2* and *Grhl3*, members of the Grainyhead-like family of transcription factors. *Grhl2* null embryos exhibit severe cranial NTDs, as well as spinal NTDs (open spina bifida)^7–9^. Notably, over-expression of *Grhl2* also causes spina bifida in the *Axial defects* (*Axd*) mouse^9^. It is not known whether spinal NTDs resulting from Grhl2 loss- and gain-of function share a common pathogenic mechanism or whether distinct requirements for closure are compromised in each model^9^.

Like *Grhl2*, loss or excess expression of *Grhl3* also causes spina bifida^6,10–13^. Moreover, mutations in *Grhl2* and *Grhl3* show additive interactions. *Grhl2/Grhl3* double knockout embryos exhibit almost complete failure of neural tube closure, with the exception of only a small region at the hindbrain-spinal boundary corresponding to the site of initial contact of the neural folds^8^. Moreover, the combined presence of over-expressing alleles of both *Grhl2* and *Grhl3* in double heterozygous embryos causes spina bifida, whereas neural tube closure proceeds to completion in each of the single heterozygous genotypes^13^. These findings highlight the crucial role of Grhl2/3 in regulating neural tube closure and the exquisite sensitivity of this process to their expression level.

Grhl2 is required for maintenance of epithelial properties in a variety of contexts, including renal epithelia, bronchial cells and liver progenitors, where it directly regulates expression of *Cdh1* and *Cldn4*^7,14–16^. Consistent with this function, Grhl2 plays a role in regulation of epithelial-mesenchymal transition (EMT). Lower expression is associated with acquisition of more mesenchymal characteristics and motility in cancer and epithelial cell lines^17–19^. Conversely, over-expression inhibits motility and invasion of gastric cancer derived cell lines^20^. Enhanced EMT may therefore underlie the association of low GRHL2 expression with various cancers^21^.

In neurulation stage embryos, *Grhl2* is expressed in the surface ectoderm but not the neuroepithelium^7,9^. In the context of cranial NTDs, *Grhl2* loss of function, as in other tissues, is associated with diminished expression of characteristic epithelial genes such as *Cdh1* (encoding E-cadherin) in the ENU-induced *Grhl2*^1*Nisw*^ strain^22^. In accordance with evidence from cell lines, it was hypothesised that these abnormalities result from impaired suppression of EMT^19^. Whether and how these surface ectoderm defects prevent cranial closure is still unknown, as is the causative mechanism underlying Grhl2-related spina bifida. In epithelia, altered expression of cell–cell junction proteins, such as E-cadherin, could potentially modulate properties of the actomyosin network to which they are coupled^23^. While such interactions influence mechanical properties of monolayers and developing epithelia in non-vertebrate systems^23,24^, a potential requirement in mammalian neural tube closure has not been investigated.

In the current study, we investigate the mechanism by which Grhl2 regulates spinal neurulation, using loss- and over-expression mouse models. We propose that disruption of biomechanical properties at the cellular level compromises integration of forces in the surface ectoderm and thereby prevents closure.

## Results

### Insufficient or excess Grhl2 expression inhibits spinal neurulation

**Fig. 1:**
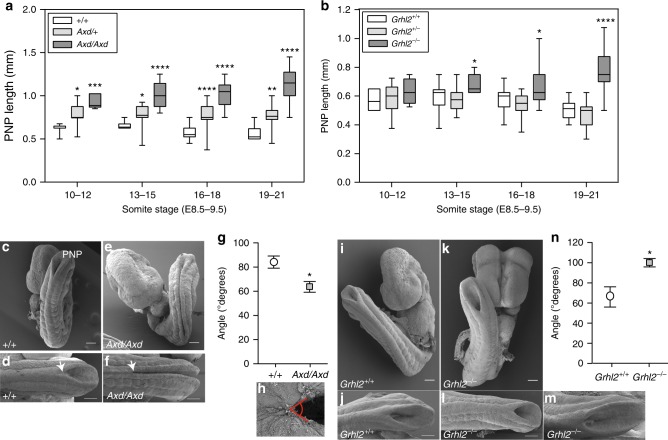
Excess or lack of Grhl2 prevents PNP closure with differing PNP morphology at developmental stages from E8.5-9.5. **a** The PNP length of *Axd/Axd* and *Axd/+* embryos was significantly enlarged compared with +/+ embryos (*n* = 215 embryos; 6–43 per genotype per stage; Supplementary Table 1). **b** The PNP length of *Grhl2*^*−/−*^ embryos was enlarged from the 13–15 somite stage (E9.0) onwards, whereas *Grhl2*^*+/−*^ embryos did not differ from wild types (*n* = 307 embryos; 4–55 per genotype per stage; Supplementary Table 1). ****p < 0.0001, ***p < 0.001, **p < 0.01, *p < 0.05 (ANOVA). **c**–**f** SEM of E9.5 embryos (18–19 somites) shows that PNP of *Axd/Axd* embryos is enlongated and very narrow compared with the PNP of wild-type embryos (**d**, **f**; arrows indicate rostral limit (closure point) of PNP). **i**–**m** The enlarged PNP of *Grhl2*^*−/−*^ embryos is characterised by widely spaced neural folds at E9.5 (examples have 20–21 somites). Consequently, the angle between the open neural folds (shown in **h**) is significantly smaller in *Axd/Axd* (**g**) and greater in *Grhl2*^*−/−*^ (**n**) embryos than in +/+ (*p < 0.05; *n* = 4–8 per genotype; Data represents mean ± SEM). Wild-type embryos did not differ between strains (**g**, **n**). Scale bars: 100 μm. Source data are provided as a Source Data file

Spina bifida in *Grhl2* null or over-expressing embryos (*Grhl2*^*−/−*^ or *Grhl2*^*Axd/Axd*^) results from failed closure of the posterior neuropore (PNP). This is preceded by the appearance of an enlarged PNP at E10.5 compared with wild-type embryos, in which closure is progressing to completion^9^. We determined the developmental stage at which PNP closure is first defective by measuring the PNP length of embryos collected during spinal closure at E9–10. A significant difference in the PNP length between *Axd/Axd* mutant and +/+ embryos is already apparent at the 10–12 somite stage (Fig. 1a), at least a day earlier than previously reported^9^. Similarly, among *Grhl2*^*−/−*^ embryos the mean PNP length is significant enlarged by the 13–15 somite stage (Fig. 1b). Hence, we focussed subsequent analysis at E9–9.5, corresponding with the onset of neurulation defects. *Axd/+* embryos also exhibit enlarged PNPs at all stages (Fig. 1a) but spinal neural tube closure, although delayed, is completed by E11.5, often with an accompanying tail flexion defect^9^. In contrast, no closure defects are apparent in *Grhl2*^*+/−*^ embryos (Fig. 1b).

Although spinal NTDs occur in both *Grhl2* over-expressing and deficient embryos, the morphology of the enlarged PNP differs between models. Scanning electron microscopy (SEM) reveals a very narrow appearance of the enlarged PNP in *Axd*/*Axd* embryos at E9.5. (Fig. 1c–f), such that the angle between the open neural folds at the closure point is smaller in *Axd/Axd* embryos than in wild types (Fig. 1g–h and Supplementary Fig. 1a). Bending of the neural folds at dorsolateral hinge points (DLHP) is required for closure in the mid-low spine^1^, but closure is already defective in *Axd/Axd* before the stage that DLHPs are required (~15 somite stage onwards), ruling out a lack of dorsolateral bending as a primary cause of spinal NTDs. At E9.5, DLHPs are present in both +/+ and *Axd/Axd* (Supplementary Fig. 1c–h). Hence, failed neural tube closure in *Axd/Axd* embryos does not result from an obvious defect in elevation or apposition of the neural folds.

*Grhl2*^*−/−*^ embryos display severe cranial NTDs comprising exencephaly with split face, in which the entire cranial neural tube remains open (Fig. 1k). In the spinal region, the neural folds of *Grhl2*^*−/−*^ mutants do not become closely opposed, unlike in *Axd/Axd* embryos (Fig. 1k–m). Instead, the PNP morphology varies; more severely abnormal embryos exhibit widely spaced neural folds with a poorly defined ‘zippering point’ (Fig. 1m), whereas other embryos have an intermediate phenotype with a distinguishable ‘zippering point’ more closely resembling wild-type embryos. Nevertheless, among *Grhl2*^*−/−*^ embryos whose PNP length is only slightly enlarged (Fig. 1k–l), the angle between the neural folds is significantly greater in *Grhl2*^*−/−*^ than in *Grhl2*^*+/+*^ embryos (Fig. 1n and Supplementary Fig. 1b), suggesting a possible defect in bringing together or stabilising the neural folds.

### Grhl2 regulates an epithelial molecular signature

**Fig. 2:**
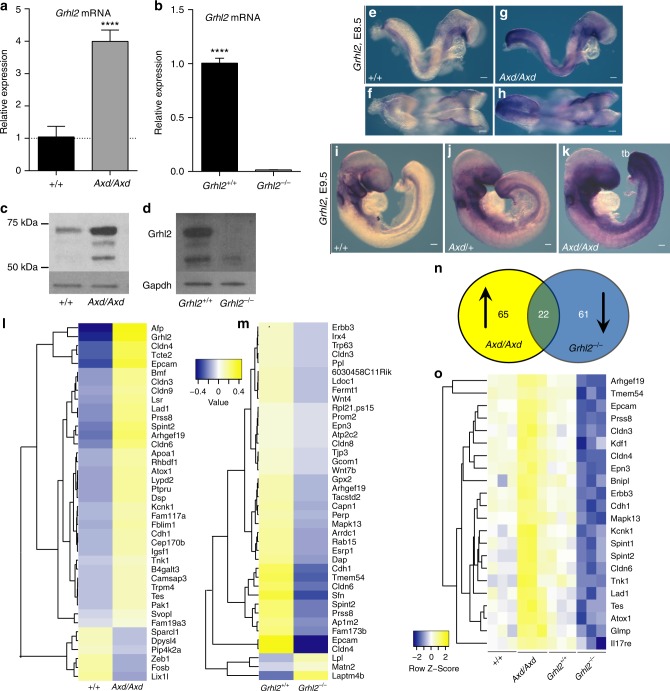
Transcriptomic effect of Grhl2 loss and over-expression. Abundance of **a**, **b** *Grhl2* mRNA and **c**, **d** Grhl2 protein is significantly elevated in the caudal region of **a**, **c** *Axd/Axd* embryos and diminished in **b**, **d** *Grhl2*^*−/−*^ embryos at E9.5 (17–19 somite stage); ****p < 0.0001; *t*-test; mRNA data represents mean ± SEM (*n* = 6 samples/genotype). Example immunoblot in **d** is exposed to show all +/+ bands. **e**–**k** Increased expression of *Grhl2* in *Axd/Axd* embryos at E8.5 (**e**, **f**; dorsal view in **f** and **h**) and E9.5 (**i**–**k**; examples at 16 somite stage). Apparent upregulation or ectopic expression is detected in the tail bud (tb; **i**–**k**) in *Axd/Axd* embryos. Scale bars represent 100 μm. Images are representative of a minimum of three embryos per genotype. **l**–**o** Heat maps of the 40 most significant differentially expressed genes in RNA-Seq analysis of the caudal region of **l** *Axd/Axd* or **m** *Grhl2*^*−/−*^ embryos and their wild-type littermates at E9.5. Boxes represent mean of triplicate samples. *Grhl2* over-expression predominantly causes upregulation (yellow shading in **l**), whereas loss of *Grhl2* causes downregulation (blue shading in **m**). **n** Among genes upregulated in *Axd/Axd* and downregulated in *Grh2*^*−/−*^, 22 were common. **o** Heat-map of the expression profile of the 22 common genes. Boxes represent individual samples. Source data are provided as a Source Data file

Quantitative reverse transcription PCR (RT-PCR) confirmed that *Grhl2* mRNA abundance in the PNP region at E9.5, is elevated in *Axd/Axd* and diminished in *Grhl2*^*−/−*^ embryos (Fig. 2a–b). In dissected PNP regions of wild-type embryos, several isoforms of Grhl2 protein are detected by immunoblot, as reported for human GRHL2^18^, and their abundance correlates with excess or diminished mRNA in *Grhl2* mutants (Fig. 2c–d). At E8.5 and E9.5, as at later stages^7,9^, *Grhl2* is expressed in the surface ectoderm and hindgut, with detectable over-expression in Axd/+ and Axd/Axd embryos (Fig. 2e–k and Supplementary Fig. 2).

Transcriptomic consequences of Grhl2 loss- and over-expression in the spinal region were investigated by RNA-sequencing (RNA-seq) using isolated caudal tissue (encompassing the PNP) from *Axd/Axd* and *Grhl2*^*−/−*^ embryos, together with their wild-type littermates at E9.5 (Fig. 2l–m). Among genes that show differential expression in *Axd/Axd* embryos compared with wild type, the majority (87 of 112) are upregulated (40 most significant are listed in Fig. 2l). In contrast, loss of *Grhl2* leads principally to downregulation of genes (83 of 113 significantly different transcripts) in *Grhl2*^*−/−*^ embryos (Fig. 2m). These datasets share 22 common genes whose expression is reciprocally dysregulated: i.e., upregulated in *Axd/Axd* and downregulated in *Grhl2*^*−/−*^ (Fig. 2n, o). *Grhl2* responsive transcripts are enriched for genes that are characteristic of epithelial cells^25^, encoding proteins that include E-cadherin, claudins and other tight junction proteins (Tjp3/ZO-3, cingulin), EpCAM, membrane-bound serine proteases/inhibitors (prostasin, spint1, spint2) and desmosome proteins (e.g., Dsp/desmoplakin, Dsg2 desmoglein2 and Ppl/periplakin). Pathway analysis of the differentially expressed genes also indicates tight junctions as a main component in both sets (Supplementary Table 2). Findings in *Axd/Axd* and *Grhl2*^*−/−*^ spinal region are consistent with previous observations of reduced expression of *Cdh1* and other epithelial genes resulting from *Grhl2* loss-of-function in other systems^7,22^.

**Fig. 3:**
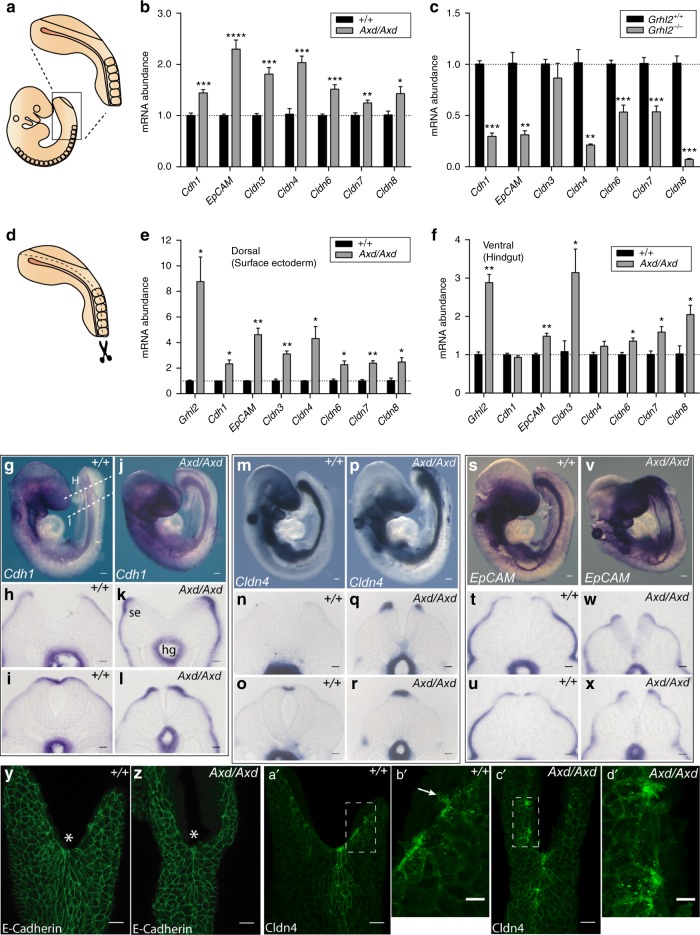
*Grhl2* regulates a molecular signature of epithelia in the surface ectoderm. **a** Schematic representation of an E9.5 embryo and caudal spinal region isolated for analysis. **b**–**c** qRT-PCR of *Cdh1, EpCAM, Cldn 3,4,6,7,* and *8* of samples derived from the whole-caudal spinal region (as in **a**) (*n* = 3–6 samples per genotype). **d** Schematic representation of the microdissection separating the dorsal (surface-ectoderm-containing) and the ventral (hindgut-containing) parts of the caudal spinal region. **e**, **f** qRT-PCR analysis of *Cdh1, EpCAM, Cldn 3,4,6,7,* and *8* of samples derived from microdissected spinal regions (as in **d**) ( > 5 embryos pooled per sample; three samples per genotype). Data represent mean ± SEM (****p < 0.0001, ***p < 0.001, **p < 0.01, *p < 0.05; *t*-test); (See Supplementary Fig. 3 for equivalent *Grhl2*^*−/−*^ data). WMISH confirmed upregulation of *Cdh1* (**g**–**l**), *Cldn4* (**m**–**r**), and *EpCAM* (**s**–**x**) in *Axd/Axd* compared with +/+ embryos at E9.5 (examples at 17–19 somite stage). Transverse sections are at the levels indicated by dashed lines in **g** (se, surface ectoderm; hg, hindgut). Scale bars represent 100 µm in whole mounts and 25 µm on sections. **y**–**d**ʹ Dorsal view of PNP region (caudal end oriented upwards; asterisk indicates closure site) immunostained for E-cadherin (**y**, **z**) and Cldn4 (**a**ʹ–**d**ʹ). **b**ʹ and **d**ʹ show magnified images of areas boxed in **a'** and **c'**, respectively. Scale bars indicate 25 μm in **y**, **z**, **a**ʹ, **c**ʹ and 10 μm in **b**ʹ and **d**ʹ. Images are representative of a minimum of three embryos per genotype. See also Supplementary Fig. 4 for additional WMISH of *Cldn* genes in *Axd* and Supplementary Fig. 5 for equivalent data in *Grhl2*^*−/−*^. Source data are provided as a Source Data file

The altered expression of a series of Grhl2 responsive epithelial genes, including *Cdh1*, *Cldn*3-8 and *EpCAM*, was confirmed by qRT-PCR analysis of the caudal region from embryos at E9.5 (17–19 somite; Fig. 3a–c). To discriminate the effect of Grhl2 in different tissues, the PNP region was microdissected (Fig. 3d–f), to separate surface ectoderm-containing dorsal tissue from hindgut-containing ventral tissue (validated by differential expression of the endoderm-specific gene, *Sox17*, Supplementary Fig. 3a–c). In *Grhl2*^*−/−*^ embryos, most differentially expressed genes are downregulated in both surface ectoderm and hindgut-containing samples (Supplementary Fig. 3d–e). Similarly, the majority of genes examined in *Axd/Axd* embryos are upregulated in both compartments, but *Cdh1* and *Cldn4* appeared to be principally upregulated in the dorsal (surface ectoderm-containing) tissue without significant difference compared with wild type in ventral, hindgut-containing samples (Fig. 3e–f).

### Grhl2 regulates the composition of apical junction complexes

Whole-mount in situ hybridisation confirmed expression of *Cdh1*, *Cldn4* and *EpCAM* in the surface ectoderm in the spinal region (Fig. 3g–x and Supplementary Fig. 4). *Cdh1* and *Cldn4* mRNA is especially abundant in the surface ectoderm overlying the neuroepithelium of the neural folds and the midline of the immediately closed neural tube (Fig. 3g–r). This enrichment is apparent in wild-type embryos and even more notable in *Axd/Axd* littermates (Fig. 3l, r). Immunostaining for E-cadherin and Cldn4 in the surface ectoderm is consistent with in situ hybridisation data and particularly evident in the midline surface ectoderm, immediately rostral to the closure point (Fig. 3y–d'). Cldn4 is localised as expected at cell borders, with prominent staining at the neuroepithelium/surface ectoderm border of the neural folds. This is especially noticeable in *Axd/Axd* embryos (Fig. 3b', d').

Expression of *Cldns* 3, 6 and 7 is upregulated in the surface ectoderm of *Axd/Axd* mutants (Supplementary Fig. 4i–n), while *Cldn8* is upregulated only in the hindgut at the level of the PNP (Supplementary Fig. 4o, p). Hence, genes encoding components of adherens junctions and tight junctions are upregulated in the surface ectoderm at the tips of the neural folds coincident with failure of progression of closure.

Although *Axd/Axd* embryos exhibit apparently ectopic expression of *Grhl2* in the tail bud (Fig. 2k and Supplementary Fig. 2c), we did not detect abnormal expression of the transcriptionally regulated targets *Cdh1* or *Cldn4* in these sites (Fig. 3k, q). *EpCAM* expression is upregulated in the tail bud and the PNP neuroepithelium (Fig. 3v and Supplementary Fig. 4f), but EpCAM protein is only detected in the surface ectoderm and not in the ectopic sites of mRNA expression (Supplementary Fig. 4h). Altogether these data suggest that NTDs caused by *Grhl2* over-expression are due to abnormalities in the normal *Grhl2* expression domain. Overall, the narrow PNP morphology, timing of PNP closure defects, and sites of expression of *Grhl2* targets (Fig. 3), indicate that the primary defect in *Axd/Axd* embryos is localised to the surface ectoderm and not the neural plate or hindgut.

### Grhl2 loss alters molecular identity of surface ectoderm

In *Grhl2*^*−/−*^ embryos, *Cdh1* mRNA expression is detectable in the surface ectoderm at the tips of the spinal neural folds, but at much lower intensity than in wild types (Supplementary Fig. 5a, b), as found in the cranial surface ectoderm and foregut^7^. Similarly, expression of *Cldn4*, *6* and *7* is very weak or undetectable in the surface ectoderm (Supplementary Fig. 5e, f, k–n) while EpCAM is absent (Supplementary Fig. 4g, h).

**Fig. 4:**
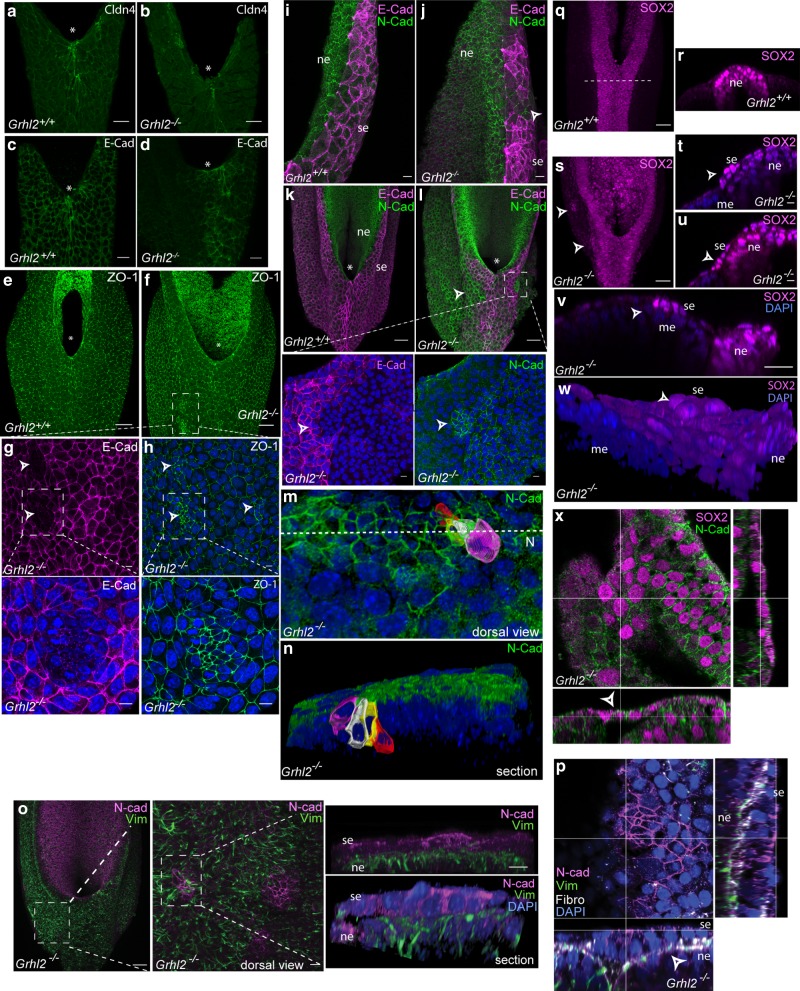
Altered regulation of epithelial and neuropeithelial markers. **a**–**d** Dorsal views of spinal region (asterisk indicates PNP closure site) with whole-mount immunostaining in stage-matched embryos at E9.5 for **a–b** Cldn4, **c**, **d** E-cadherin. **e**, **f** Immunostaining for ZO-1 in the spinal region at E9.5. **g**, **h** Magnification of the boxed area in **f**, showing clusters (arrowheads) that are negative for E-cadherin (magenta) and positive for ZO-1 (green) (nuclei stained blue with DAPI). **i**–**l** Surface-subtracted images of N-cadherin (green) and E-cadherin (magenta) staining, showing upregulation of N-cadherin expression in the surface ectoderm (se) of *Grhl2*^*−/−*^ mutants (arrow in **j**, **l**). Boxed area in **l** indicates an N-cadherin (green) positive/E-cadherin (magenta) negative cluster (indicated by arrowheads in magnified views; nuclei stained blue with DAPI). **m** Dorsal and reconstructed **n** view of N-cadherin-positive cells in a cluster in the surface ectoderm layer of a *Grhl*^*−/−*^ embryo. **o** Immunostaining for vimentin (green) in the basal neuroepithelium (ne), including underlying an N-cadherin-positive (magenta) cluster in the surface ectoderm (also shown in sections). **p** An N-cadherin-positive cluster separated by fibronectin (white) from underlying, vimentin-positive neuroepithelium. **q**–**x** Immunostaining for Sox2 in the caudal region of wild-type (**q**–**r**) and *Grhl2* null (**s**–**x**) embryos at E9.5. Sox2-positive cells are observed in the surface ectoderm (se) layer in *Grhl2* null embryos (arrows in **s**–**x**) but not in wild types (**q**–**r**). Images are representative of a minimum of three embryos per genotype. Scale bars represent 25 µm (**a**–**d**, **v**), 50 µm (**e**–**f**, **k**–**l**, **o**, **q**, **s**) and 10 µm (**i**–**j**, **r**, **t**–**u**)

As predicted by mRNA expression, Cldn4 immunostaining is diminished in the surface ectoderm of *Grhl2*^*−/−*^ embryos compared with wild-type controls (Fig. 4a, b). Weak E-cadherin staining is detectable in the midline of *Grhl2*^*−/−*^ embryos (Fig. 4c, d and Supplementary Fig. 5c, d). In other areas of the surface ectoderm E-cadherin staining is very weak or absent (Fig. 4d, g, l). However, this cell layer appears to be intact based on expression of other components of adherens junctions (*Ctnnb1*; β-catenin) and tight junctions (*Tjp1*; ZO-1) (Supplementary Fig. 6). *Ctnnb1* and *Tjp1* mRNA expression do not differ between *Grhl2*^*−/−*^ and their wild-type littermates (Supplementary Fig. 6a, b), while localisation of the corresponding proteins in the surface ectoderm has an overall normal appearance (Fig. 4e, f and Supplementary Fig. 6).

In wild-type embryos there is a clear demarcation of N-cadherin-positive neuroepithelium and E-cadherin-positive surface ectoderm (Fig. 4i, k). However, in E-cadherin deficient areas of *Grhl2* null surface ectoderm, we observe ectopic N-cadherin (arrows in Fig. 4j, l). Within these N-cadherin-positive regions, some E-cadherin-negative/N-cadherin-positive cells form discrete ‘clusters’ (boxed region in Fig. 4l). These E-cadherin-negative clusters exhibit dense ZO-1 staining (Fig. 4g, h), suggesting constriction of the apical borders (where ZO-1-positive tight junctions are located) of these cells in contrast to the usual flattened appearance of the squamous surface ectoderm in surrounding areas. Consistent with this observation, 3D-reconstruction of in silico*-*segmented cells shows that N-cadherin-positive cells within the surface ectoderm cell clusters adopt wedged shapes that are characteristic of a pseudo-stratified epithelium (like the neuroepithelium) and not of a squamous surface ectoderm (Fig. 4m, n).

Acquisition of N-cadherin and downregulation of E-cadherin are hallmarks of EMT^26^. In epithelial and cancer cell lines, Grhl2 is required for maintenance of epithelial phenotype and regulation of EMT^21,27^. This is mediated both by regulation of epithelial genes such as *Cdh1* and reciprocal repressive interaction with the pro-mesenchymal regulator, Zeb1^7,17,18,27^. This raised the question of whether partial EMT may contribute to spinal NTDs in *Grhl2*^*−/−*^ embryos. Arguing against this idea, we do not observe upregulation of *Zeb1*, *Zeb2* or *vimentin* mRNA in the surface ectoderm of the spinal region of *Grhl2*^*−/−*^ embryos (Supplementary Fig. 7). Although having a predominantly mesenchymal localisation, vimentin is not a definitive mesenchymal marker being also detected in the basal neuroepithelium (Supplementary Fig. 7) as reported previously^28^. Immunostaining does reveal the abnormal presence of vimentin-positive cells in the ventral hindgut (arrow in Supplementary Fig. 7n), suggesting a requirement for Grhl2 to maintain cell identity in this tissue. Consistent with findings in the cranial region^19^, we note an enrichment of vimentin staining at the closure point of the neural folds in some *Grhl2*^*−/−*^ (5 of 7 embryos), which appears to be subjacent to the E-cadherin-positive surface ectoderm (Supplementary Fig. 7n). Vimentin staining is not enriched in N-cadherin-positive clusters in the surface ectoderm (Fig. 4o) but is present in the underlying neuroepithelium (Fig. 4o, p), the border being delineated by fibronectin-containing basement membrane (Fig. 4p).

Overall, with respect to current views of epithelial identity as a continuum with a spectrum of intermediate phenotypes (e.g., in respect to EMT)^29^, our findings are consistent with a transition towards a less epithelial state in surface ectoderm of *Grhl2*^*−/−*^ embryos, but not EMT. N-cadherin is not only a mesenchymal marker but also the cadherin expressed in the neuroepithelium, a pseudo-stratified epithelium. Hence, we could not rule out the possibility that N-cadherin-positive clusters observed in the *Grhl2* null surface ectoderm have acquired a more neuroepithelial rather than mesenchymal character. We therefore examined the neuroepithelium-specific marker, Sox2 (Fig. 4q–x), which is not expressed in the surface ectoderm of wild-type embryos (Fig. 4q, r). Notably, the surface ectoderm of *Grhl2* null embryos contains Sox2-positive cells (Fig. 4s–v), with flat or columnar morphology (Fig. 4w). Sox2-positive cells co-label with N-cadherin (Fig. 4x) and can be found overlying the neuroepithelium, the mesenchyme or at the border between the two (Fig. 4s–v). These findings suggest that loss of epithelial identity in *Grhl2* null surface ectoderm is associated with gain of neuroepithelial character.

In *Grhl2* over-expressing (*Axd/Axd*) embryos, expression of *Ctnnb1* (β-catenin) and *Tjp1* (ZO-1) does not differ from wild-type littermates (Supplementary Fig. 6e), the surface ectoderm is intact and does not express N-cadherin (Supplementary Fig. 7w). Upregulation of characteristic ‘epithelial’ genes, in the surface ectoderm of *Axd/Axd* embryos, raises the question of whether mesenchymal markers are reciprocally regulated in these mutants. Interrogation of our RNA-seq data and qRT-PCR analysis, reveals downregulation of the mesenchymal markers *vimentin*, *Zeb1* and *Zeb2* in the spinal region of *Axd/Axd* embryos (Fig. 2l and Supplementary Fig. 7b). In accordance with this finding, vimentin mRNA and protein show reduced abundance in the mesenchyme of the caudal region, where *Grhl2* is expressed in *Axd/Axd* embryos (Supplementary Fig. 7q–v).

Our observations suggest that the surface ectoderm of *Grhl2* over-expressing (*Axd/Axd*) embryos exhibits a shift towards a ‘super-epithelial’ character, as opposed to the shift towards a less epithelial and more neuroepithelial character in *Grhl2*^*−/−*^ embryos.

### Grhl2 dysregulation causes abnormal actomyosin distribution

Despite the differential effect on surface ectoderm identity, neither loss- nor over-expression of *Grhl2* mutants affected the presence of the characteristic membrane ruffles, which emanate from surface ectoderm cells and are required for spinal closure^3^ (Supplementary Fig. 8). We therefore investigated other mechanisms by which altered surface ectoderm properties in *Grhl2* mutants could compromise neural tube morphogenesis.

In the spinal region of *Grhl2*^*−/−*^ embryos, the detectable E-cadherin is generally most abundant in the midline (reflecting the wild-type distribution), whereas the areas of absent E-cadherin/high N-cadherin/abnormal ZO-1 expression, are not observed at the closure site of the neural folds (Fig. 4). This suggests that these clusters are not directly responsible for failure of closure in a cell autonomous manner. Nevertheless, altered composition of the AJCs suggests a mechanism by which surface ectoderm properties could be altered via modulation of the cytoskeleton. Actomyosin network activity modulates cell and tissue behaviours in a variety of developmental contexts, a property that depends on coupling to the plasma membrane and between cells via adherens and tight junctions^30–32^. For this reason, we examined F-actin and phospho-myosin light chain II (p-MLCII) in *Grhl2* null and over-expressing embryos.

**Fig. 5:**
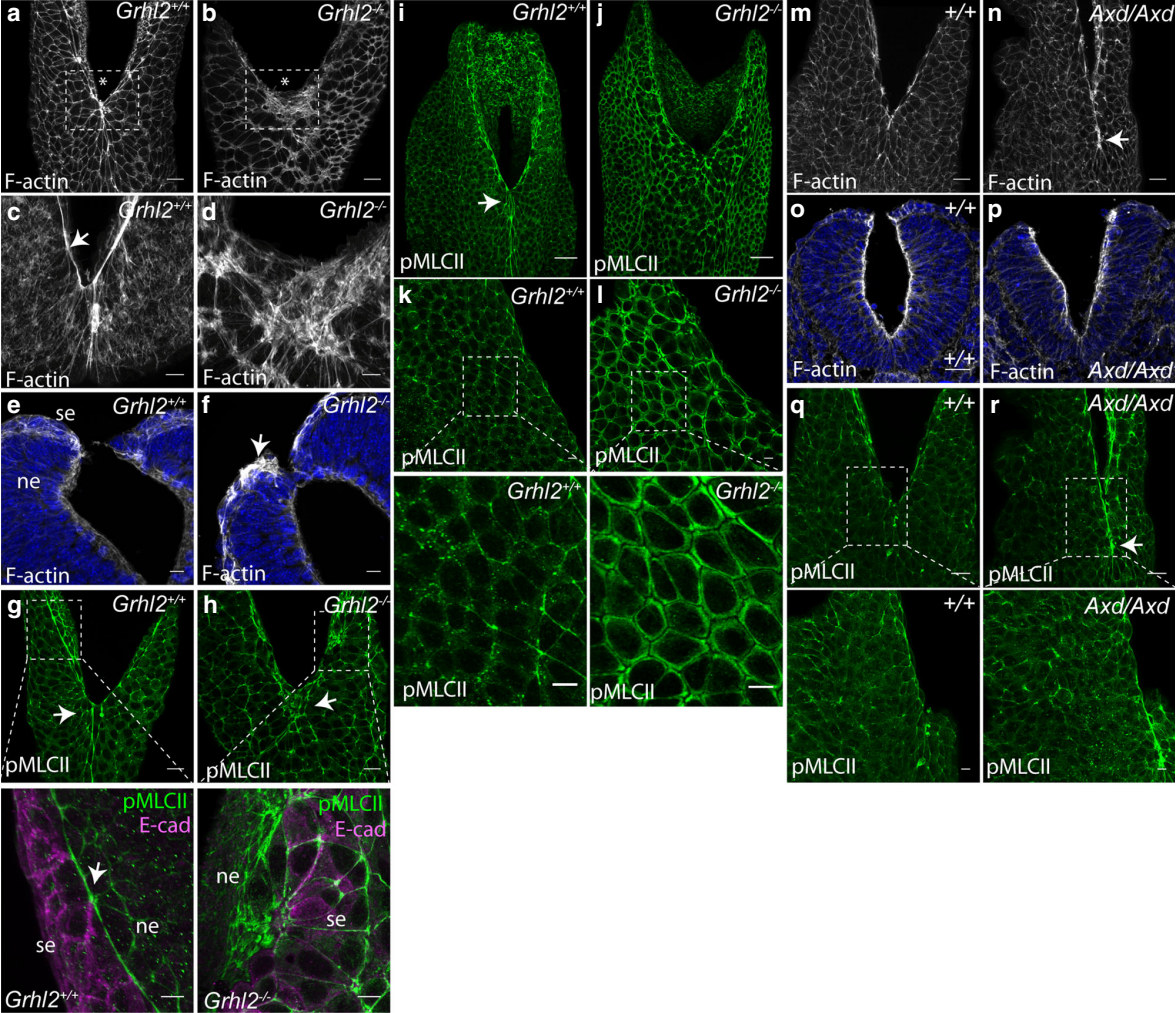
Cytoskeletal abnormalities in surface ectoderm of Grhl2 mutant embryos. **a**, **b**, **m**, **n** Surface-subtracted images of dorsal view of phalloidin stained PNP regions (asterisk indicates closure point) of *Grhl2*^*−/−*^ and *Axd/Axd* mutants and equivalent wild types at E9.5. F-actin localises at cell boundaries in the surface ectoderm of wild-type controls (**a**, **m**), and shows profoundly disturbed organisation in *Grhl2*^*−/−*^ (box in **b**) and greater intensity of staining in the midline in *Axd/Axd* (arrow in **n**). **c**, **d** High-resolution images of the PNP closure point (boxed in **a**, **b**), show enrichment in an ‘actin cable’ at the margins of the open neural folds in wild type (arrow in **c**), which is disrupted in *Grhl2*^*−/−*^ (**d**). **e**, **f**, **o**, **p** Transverse sections of phalloidin (grey) stained *Grhl2*^*−/−*^ and *Axd/Axd* embryos at E9.5 (nuclei stained blue with DAPI). Disturbed F-actin organisation in the surface ectoderm at the PNP closure point in *Grhl2*^*−/−*^ embryos is indicated (arrow in **f**). **i**–**l** Immunostaining for p-MLCII (green) at E9.5. In wild-type embryos, surface-subtracted images of dorsal views of the PNP show notable enrichment in a ‘cable’ at the surface ectoderm (se)/neuroepithelium (ne) boundary in the PNP (arrow in magnified image in **g**), as well as in the midline overlying the most recently closed neural tube (arrows in **g**, **I**). Like F-actin, pMLCII is disorganised in *Grhl2*^*−/−*^ embryos at the PNP closure point (arrow in **h**) and at discrete regions of the open neural folds (boxed in **h**). In contrast to pMLCII localisation at cell–cell junctions in the surface ectoderm of wild-type embryos (**k**, **q**), pMLCII appeared uniformly distributed around the margins of surface ectoderm cells and excluded from cell–cell junctions in *Grhl2*^*−/−*^ embryos (**l**). **m**–**r** The localisation of F-actin (**m**–**p**) and pMLCII (**q**, **r**) in *Axd/Axd* was comparable to wild-type embryos at E9.5 but consistently exhibited greater intensity of staining in the midline (arrows in **n** and **r**). Scale bars: 10 μm in **c**, **d**, **k**, **l** and magnified areas of **g**–**h**, **k**–**l**, **q**, **r**; 25 μm in **a**, **b**, **g**, **h**, **m**, **n**, **q**, **r**; 50 μm in **i**, **j**. Images are representative of a minimum of three embryos per genotype

Phalloidin staining reveals F-actin enrichment at the margin of the neural folds in wild-type embryos (Fig. 5a, arrow in **c**). This corresponds to a rostro-caudally oriented F-actin cable, which is localised to the tips of the neural folds along the margin of surface ectoderm and neural plate, and which comes to encircle the open PNP by the latest stages of spinal neurulation^33^. In contrast, the closure point of *Grhl2*^*−/−*^ embryos frequently exhibits a disorganised mesh of F-actin fibres localised in the surface ectoderm (Fig. 5b, d, arrow in **f**).

In wild-type embryos, immunostaining for p-MLCII shows enrichment in the midline overlying the recently closed neural tube (arrows in Fig. 5g, i). At the margin of the open neural folds, p-MLCII staining has the appearance of a continuous ‘cable’, similar to F-actin (boxed area in Fig. 5g). Co-staining with E-cadherin shows that this lies at the border of surface ectoderm and neuroepithelium in the neural folds of wild-type embryos (magnified area in Fig. 5g). *Grhl2*^*−/−*^ embryos exhibit striking abnormalities in p-MLCII localisation, with disorganisation or absence of a distinct ‘cable’ at the neural fold margins (Fig. 5h). As for F-actin, pMLCII staining in the midline overlying the recently closed neural tube reveals regions of intense staining, with a disorganised arrangement of fibres (arrow in Fig. 5h).

In the surface ectoderm itself, p-MLCII is enriched at cell–cell junctions in wild-type embryos (Fig. 5k), whereas in *Grhl2*^*−/−*^ embryos pMLC-II appears to be excluded from cell borders with a uniform cortical localisation (Fig. 5l). Hence, loss of epithelial character in *Grhl2* null embryos is associated with a significant disturbance of the actomyosin distribution, both at the closure point and throughout the surface ectoderm. These findings suggest that the absence of Grhl2 may be associated with disturbance of biomechanical properties in the surface ectoderm layer.

In contrast to *Grh2* null embryos, staining for F-actin revealed a similar distribution in *Axd/Axd* and +/+ embryos (Fig. 5m–r). However, on whole-mount phalloidin immunostaining, *Axd/Axd* embryos consistently showed more intense F-actin staining than wild-type littermates at the margins of the neural folds and midline of the closed neural tube (arrow in Fig. 5n). Similarly, compared with +/+ littermates, the abundance of p-MLCII appeared greater in the surface ectoderm at the midline of *Axd/Axd* embryos, but with normal localisation at cell–cell junctions in the surrounding surface ectoderm (Fig. 5q–r). Enriched actomyosin abundance at the midline suggests that biomechanical properties of the surface ectoderm may be altered in this region.

### Grhl2 affects surface ectoderm cell shape and thickness

**Fig. 6:**
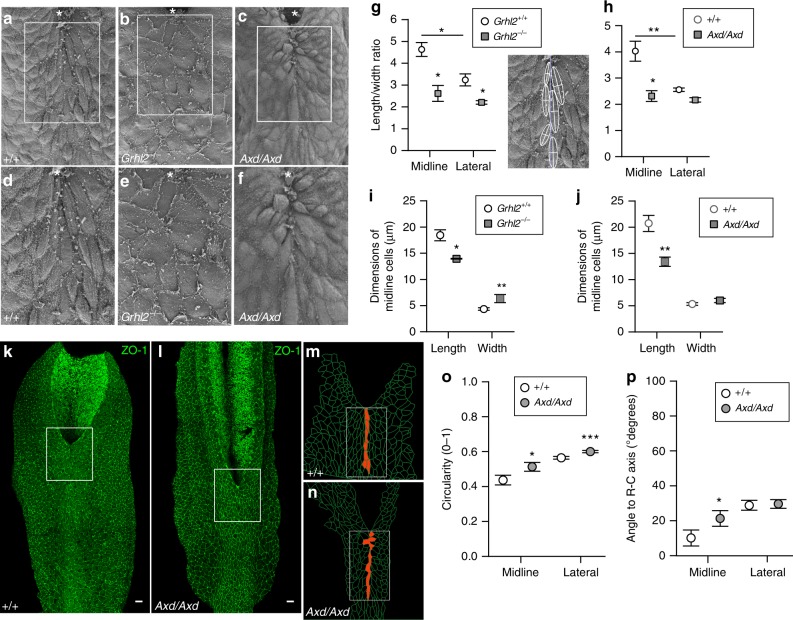
*Grhl2* dysregulation leads to abnormal surface ectoderm cell shape. **a**–**c** Dorsal SEM images of the spinal region (oriented with caudal to top) at the PNP closure point (indicated by asterisk) of wild-type, *Grhl2*^*−/−*^ and *Axd/Axd* embryos at E9.5 (examples have 21 somites). **d**–**f** Magnified view of areas boxed in **a**–**c**, respectively. **g**, **h** Quantification of the length/width ratio of surface ectoderm cells located in the midline (based on major and minor axes as shown superimposed in inset image) and the lateral cells (remaining cells in the box) in *Grhl2*^*−/−*^ and *Axd/Axd* mutants and corresponding wild-type controls. Wild-type embryos for both strains show significant change of length/width ratio between midline and lateral cells (**p < 0.01, *p < 0.05; *t*-test). Number of cells analysed = 19–102 per group; 3–5 embryos per genotype (see Supplementary Table 3). **i**, **j** Dimensions of cells in the midline. Data represent mean ± SEM (significant difference from wild type in equivalent region, **p < 0.01, *p < 0.05; *t*-test). **k**, **l** Cell outlines were segmented (using tissue analyser) from dorsal images of whole-mount ZO-1 immunostaining of the PNP region of *Axd/Axd* and +/+ embryos at E9.5. Scale bars: 50 μm. **m**, **n** Magnified view of boxed areas in **k** and **l**. **o**, **p** Circularity and angle from the rostro-caudal axis were determined for midline cells (orange in **m**, **n**) and lateral cells (remaining cells in box in **m**, **n**) for *Axd/Axd* and +/+ embryos (*n* = 3 per genotype; 22–27 midline cells and 260–332 lateral cells per genotype; significant difference from wild type, ***p < 0.001, *p < 0.05; *t*-test). Source data are provided as a Source Data file

We next asked whether dysregulation of AJC components and perturbation of actomyosin organisation is associated with cellular abnormalities that may reflect altered propagation of forces in the surface ectoderm. E-cadherin whole-mount immunostaining in wild-type embryos (Figs. 3y and 4c, k), reveals apparent rostro-caudal elongation of midline cells in the surface ectoderm overlying the most recently closed neural tube. This was confirmed in SEMs of the dorsal surface (Fig. 6a, d), whereas in *Grhl2*^*−/−*^ and *Axd/Axd* mutants, the shapes of midline cells appeared abnormal (Fig. 6b, c, e, f), with a wider or more rounded appearance. The length-width ratio of midline cells and their lateral neighbours was determined, based on their major and minor axes after their in silico conversion to ellipses (insert in Fig. 6g). In both *Grhl2*^*−/−*^ and *Axd/Axd* mutants we observe a lower length-width ratio of midline cells, signifying less elongation than in wild types (Fig. 6g, h). In *Grhl2*^*−/−*^ embryos, the alteration in shape of midline cells results from a combination of reduced rostro-caudal length and increased medio-lateral width (Fig. 6i), whereas the altered ratio in *Axd/Axd* embryos (Fig. 6j), is principally due to diminished length implying that the cross-sectional area is smaller.

Additional analysis of cell shape in surface ectoderm of *Axd/Axd* embryos was based on segmentation of ZO-1 immunostained images (Fig. 6k, l). Consistent with reduced length-width ratio on SEM, the circularity of surface ectoderm cells (Fig. 6o) is significantly higher in *Axd/Axd* embryos than +/+ in the midline (highlighted in orange in Fig. 6m, n) and in lateral regions (boxed, excluding midline, in Fig. 6m, n). Midline surface ectoderm cells of *Axd/Axd* embryos also exhibit disturbance in their orientation with respect to the rostro-caudal axis (Fig. 6p).

**Fig. 7:**
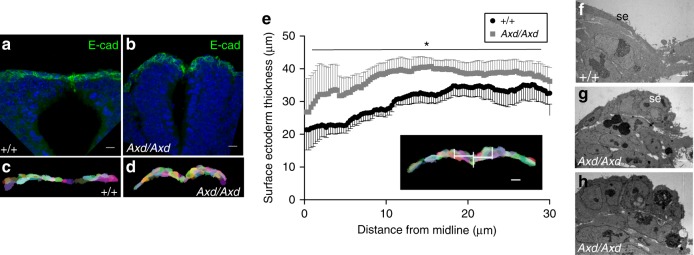
Increased thickness of surface ectoderm in *Axd/Axd* embryos. **a**–**e** Surface ectoderm cells overlying the dorsal neural tube were segmented using z-stacks from E-cadherin (green) stained transverse sections (**a**, **b**) at the PNP closure site of E9.5 embryos, using Seedwater Segmenter (**c**, **d**). Scale bars: 10 μm. **e** Thickness of the segmented surface ectoderm was determined across a 30 μm interval either side of the midline, with a measurement taken every 0.26 µm (*n* = 6 +/+, *n* = 8 *Axd/Axd*; *p < 0.001 by mixed model, thickness varies with genotype). Scale bar: 10 µm. Data are represented as mean ± SEM. **f**–**h** Representative transverse TEM images show the cuboidal phenotype of surface ectoderm (se) cells in *Axd/Axd* mutants (**g** and **h**) in comparison with the flat cells in +/+ (**f**). Scale bars: 2 µm. se: surface ectoderm. Source data are provided as a Source Data file

The observed decrease in cell length, without change in width, in the plane of the *Axd/Axd* surface ectoderm prompted examination of apical-basal thickness. Surface ectoderm cells were segmented (Fig. 7c, e), based on E-cadherin immunostaining of transverse sections through the spinal region of E9.5 embryos (Fig. 7a, b). Measurements, at 1 µm intervals either side of the midline, reveal a significantly increased thickness of the surface ectoderm at the dorsal tips of the neural folds in *Axd/Axd* embryos (Fig. 6e). Hence, over-expression of *Grhl2* results in conversion of the characteristic flattened, elongated surface ectoderm cells to a more rounded phenotype with greater apical-basal thickness, as confirmed by transmission electron microscopy (TEM) (Fig. 7f–h). These observations suggest that the excess abundance of components of adherens and tight junctions, resulting from *Grhl2* over-expression, may favour additional formation of lateral cell junctions resulting in increased apical-basal height. Altered cell dimensions also suggest that surface ectoderm cells experience altered tissue tension in *Axd/Axd* mutants, which we predict to result from a change in the balance of cell–cell junctional strength.

### A biomechanical deficit in the Grhl2 null surface ectoderm

**Fig. 8:**
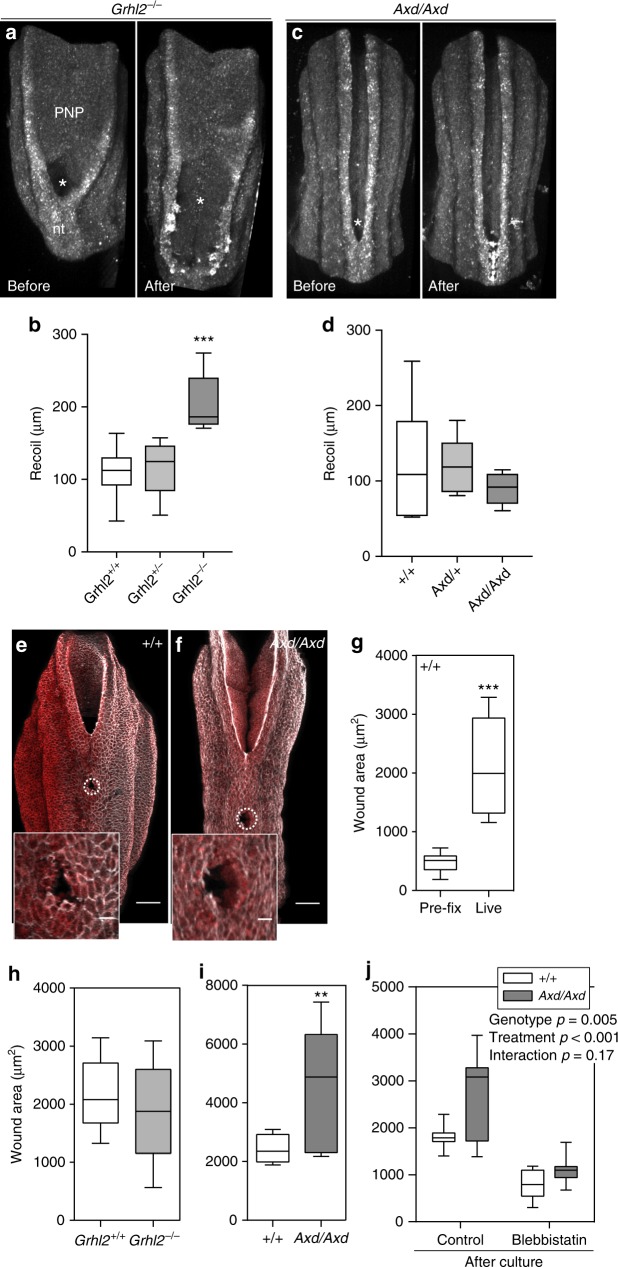
Grhl2 regulates biomechanical properties of the surface ectoderm. **a**–**d** The PNP region (oriented caudal upwards) of *Grhl2*^*−/−*^ (**a**) and *Axd/Axd* (**c**) mutant embryos at E9.5 before and after laser ablation of the neural tube (nt) closure point (indicated by asterisk). The neural folds of *Grhl2*^*−/−*^ show significantly greater recoil after ablation than other genotypes (**b**), whereas recoil in *Axd/Axd* mutants does not differ from other genotypes (**d**) (*n* = 6–9 per genotype; ***p < 0.001, one-way ANOVA). **e**, **f** Typical appearance and location of surface ectoderm puncture in the spinal region at E9.5 (rostral to the PNP closure point) following post-puncture fixation and phalloidin (grey) and CellMask^TM^ (red)-staining. Scale bar represents 100 µm and 20 µm in insert. **g** In wild-type embryos, in which surface ectoderm puncture was performed ‘live’, prior to fixation, the wound area was significantly greater than among embryos that were pre-fixed prior to puncture. **h** Surface ectoderm retraction following puncture did not differ between *Grhl2*^*+/+*^ and *Grhl2*^*−/−*^ embryos (*n* = 6/genotype), whereas **i** the puncture area was significantly greater in *Axd/Axd* embryos than among wild types (*n* = 9 per genotype; **p < 0.01, ***p < 0.001, Mann–Whitney *U-*test). **j** After 8 h in culture with vehicle, surface ectoderm retraction was greater among *Axd/Axd* than +/+ embryos as in non-cultured embryos, whereas treatment with 50 µM blebbistatin abolished this difference (*n* > 7 per genotype for both conditions; *p*-values indicate results of two-way ANOVA). Source data are provided as a Source Data file

Changes in cell shape and cytoskeletal organisation of *Grhl2* mutant embryos led us to ask whether functional dynamic properties of the surface ectoderm are disrupted on a multicellular scale. We previously found that the PNP is a biomechanically coupled structure and that ablation of the closure point leads to lateral displacement of the neural folds^33^. In order to test whether altered *Grhl2* expression in the surface ectoderm affects tissue properties of the closing neural folds we performed laser ablation of the midline roof of the immediately closed neural tube at E9.5. Retraction of the neural folds was significantly greater in *Grhl2*^*−/−*^ embryos than in wild-type littermates (Fig. 8a, b). These findings indicate that the closure point is under greater lateral tension and/or that the abnormal surface ectoderm is less able to resist retraction of the neuroepithelium resulting from ‘anti-closure’ forces in the neural folds. In contrast, average displacement of the neural folds in *Axd/Axd* embryos did not differ from wild-type embryos and the maximal recoil observed was lower than in other genotypes (Fig. 8c, d).

### Increased stress in Grhl2 over-expressing surface ectoderm

As a complementary approach, we developed a method for inferring mechanical stress withstood in the mouse embryo surface ectoderm, by adapting a technique in which stress resultants in the non-neural epidermis of *Xenopus* embryos were inferred from the expansion of microsurgical slits^34^. We made a circular stab wound, of approximately the size of one surface ectoderm cell, in the dorsal surface ectoderm of live embryos with a taper point microsurgical needle and quantified the wound area (Fig. 8e, f). The puncture was located over recently closed neural tube in order to evaluate surface ectoderm properties without ‘whole tissue’ retraction as measured with the closure point ablations.

Puncture of the surface ectoderm was followed immediately by fixation and imaging to measure ‘wound’ diameter. This reveals rapid recoil of the surface ectoderm in wild-type embryos (Fig. 8g). The method was initially validated by confirming that the resulting wound area was significantly greater in living embryos than controls, which were PFA-fixed prior to puncture (437 ± 66% bigger wound area; Fig. 8g). The recoil from the puncture site is not significantly different in Grhl2^−/−^ embryos from wild-type littermates (Fig. 8h). In contrast, the ‘wound’ size following puncture of the surface ectoderm in *Axd/Axd* mutants, is significantly larger than among wild-type littermates (Fig. 8i), suggesting that the surface ectoderm generates greater stress in *Grhl2* over-expressing embryos.

Cellular mechanical properties are regulated by actomyosin contractility^35,36^. We therefore asked whether the increased local recoil of the *Axd/Axd* surface ectoderm depends on myosin contractility. Embryos were cultured for 8 h in the presence of blebbistatin, an established inhibitor of actomyosin assembly. We used a dose which we previously found to disrupt actomyosin cross-linking^37^ without significantly affecting PNP closure of wild-type embryos^37^ (confirmed in Supplementary Fig. 9). *Axd/Axd* embryos cultured in the presence of vehicle for 8 h recapitulate the increased recoil phenotype observed in freshly dissected embryos (Fig. 8j). However, blebbistatin treatment abolishes the increased recoil of the surface ectoderm cells in the *Axd/Axd* mutants, showing that this is actomyosin-dependent. Hence, over-expression of Grhl2 alters the biomechanical properties of the surface ectoderm, most likely through raised abundance of components of the apical junction complex, and increased cell–cell forces are transmitted via the actomyosin network.

### Partial rescue of PNP closure by myosin inhibition in *Axd/Axd* embryos

**Fig. 9:**
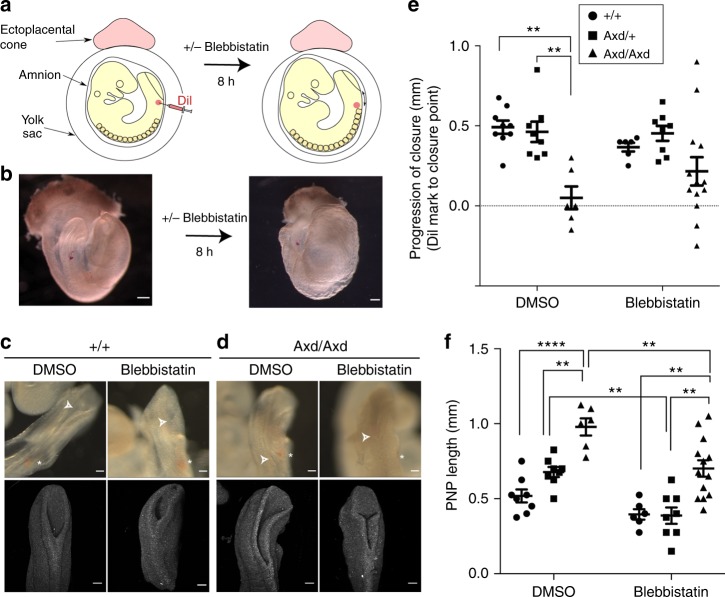
Partial rescue of PNP closure in *Axd/Axd* embryos by myosin II inhibition. **a** Diagram of experimental approach and **b** representative images of embryos, contained within the yolk sac after DiI marking and after 8 h culture (scale bars represent 500 µm). **c**, **d** Light (upper row) and confocal (lower row) images of the PNP region (caudal end oriented upwards) after culture in the presence of vehicle or 50 µM blebbistatin (effective dose is higher than in Fig. 8 as the yolk sac is open). Asterisks indicate the position of the DiI mark and arrows indicate the neural tube closure point (scale bars represent 100 µm). **e** Progression of closure is diminished in *Axd/Axd* embryos compared to other genotypes when cultured in vehicle only, but not after culture with blebbistatin. **f** PNP length is greater in *Axd/Axd* embryos than in other genotypes in both treatment groups but blebbistatin-treated mutants have a smaller PNP than vehicle-treated mutants (*n* > 6 per group; **p < 0.01, ***p < 0.0001, One-Way ANOVA). Source data are provided as a Source Data file

We hypothesise that enhanced actomyosin-mediated stress in the *Grhl2* over-expressing surface ectoderm confers resistance to neural fold retraction after laser ablation, but also impairs properties that are required for progression of ‘zippering’ of the spinal neural folds. To test this idea we asked whether blebbistatin treatment, which lowers surface ectoderm stress (Fig. 8j), had any effect on PNP closure in *Axd/Axd* embryos. Prior to culture with blebbistatin, embryos were marked with DiI adjacent to the closure point (dorsal to the mesoderm), after opening a window in the yolk sac (Fig. 9a, b). After 8 h of culture, the distance between the closure point and the DiI mark was determined to provide a measure of the progression of closure during the culture period (Fig. 9c, d). Among vehicle-treated wild-type and heterozygous embryos, PNP closure progresses ~0.5 mm during the 8 h culture period, whereas most *Axd/Axd* embryos exhibit very little or no closure (Fig. 9e). Consistent with this finding, the PNP is significantly longer among vehicle-treated *Axd/Axd* embryos than *Axd/*+ or +/+ embryos after culture (Fig. 9f). Notably, blebbistatin-treated *Axd/Axd* mutant embryos showed some progression of PNP closure, such that the distance closed during culture did not differ from other genotypes (Fig. 9e). As a result, among *Axd/Axd* embryos the PNP length was significantly smaller after blebbistatin treatment than after vehicle treatment (Fig. 9f), indicating a partial rescue of closure. *Axd/*+ embryos also exhibit a smaller PNP after culture but wild types are unaffected at this dose (Fig. 9f).

## Discussion

Spinal neural tube closure is highly sensitive to the abundance of Grhl2; with loss or over-expression of *Grhl2*, leading to spina bifida. Failure of PNP closure is apparent only a few hours after initiation of spinal neurulation and the PNP already shows significant enlargement from E9.0 among *Grhl2* null and over-expressing (*Axd/Axd*) embryos.

*Grhl2* is expressed in the hindgut as well as the surface ectoderm. We previously found that *Axd/Axd* embryos exhibit excess ventral curvature of the caudal region at E10.5, associated with diminished proliferation in the hindgut, and speculated that this could contribute to non-tissue autonomous failure of PNP closure^9^, as found in *curly tail* (*Grhl3* hypomorphic) mouse embryos and gut-specific *Grhl3* conditional knockouts^6,38^. However, this defect cannot be the cause of initial closure failure at E9.0 as excess curvature was observed ~36 h later at E10.5, supporting the hypothesis that spinal NTDs originate from a defect in the *Grhl2*-expressing surface ectoderm. Similarly, closure fails earlier in development in *Grhl3* null embryos than in gut-specific knockouts, also implicating the surface ectoderm in initial failure of closure in this model^6^. Given that neurulation fails in *Grhl2* mutants prior to onset of surface ectoderm-derived BMP regulation of neuroepithelial bending^1^, it appears that NTDs result from an abnormality within the surface ectoderm itself, involving a local defect at the PNP closure point and/or a longer-range effect of the abnormal surface ectoderm on the closure process.

Transcriptomic analysis showed that Grhl2 is a pro-epithelial transcription factor in the surface ectoderm at neurulation stages, as in other developing epithelia. The reciprocal transcriptomic effects of *Grhl2* loss or gain-of-function, as well as differing PNP morphology, suggest that differing surface ectoderm-related mechanisms underlie prevention of spinal neurulation in the two models.

In *Grhl2*^*−/−*^ embryos, the downregulation of epithelial genes, including E-cadherin and tight junction components, together with the abnormal presence of N-cadherin in the surface ectoderm layer are consistent with a transition towards a less epithelial state. However, the surface ectoderm remains as an intact cell layer (e.g., shown by immunostaining for ZO-1 and β-catenin) and E-cadherin is still detectable, particularly in the midline. Unlike in cancer cell lines^17,18^, the *Grhl2* null surface ectoderm does not appear to undergo EMT. Instead, the ectopic presence of N-cadherin indicates a shift towards neuroepithelial identity, and this is particularly evident in Sox2-positive cell clusters with columnar morphology in the surface ectoderm layer.

In epithelial tissues, adjacent cells are mechanically coupled via E-cadherin containing AJCs that are linked to the cellular contractile cytoskeleton^23,39^. Assembly of AJCs at cell–cell contacts is regulated in cultured cells by actomyosin components, including myosin IIA^40^. Conversely, E-cadherin at adherens junctions contributes to assembly of the actomyosin network, as well as modulating actomyosin properties, for example causing stiffening when force is applied to E-cadherin adhesions^23,41,42^. Our findings in *Grhl2* mutant embryos support the idea that the regulation of actomyosin by AJCs, also occurs in the developing surface ectoderm during mammalian neural tube closure. Hence, in parallel with diminished epithelial identity, Grhl2 loss of function results in profound disorganisation of an actomyosin cable, which characterises the PNP closure point of wild-type embryos (current study and ref. ^33^). Moreover, the tissue-wide actomyosin network is also disrupted with abnormal localisation of phospho-myosin. Altered cell shape in the surface ectoderm also suggests that the cells are subject to altered mechanical forces compared with wildtypes. We hypothesise that diminished abundance of adherens and tight junction proteins in the *Grhl2* null surface ectoderm is the cause of the observed actomyosin defects. Such a model is reminiscent of the effect of experimental loss of adherens junction during dorsal closure in Drosphila, which results in disruption of actin organisation and compromised epithelial integrity^43^.

The molecular and cellular effects of *Grhl2* over-expression are largely opposite to those of *Grhl2* loss of function, yet remarkably also result in severe spina bifida. Hence, excess *Grhl2* reinforces the epithelial state of the surface ectoderm in *Axd/Axd* embryos. It is predicted that, in common with other epithelia^23,35,44^, force propagation through the surface ectoderm depends on cell–cell linkage of the contractile cytoskeletal machinery via AJCs. Our findings suggest a model in which upregulation of AJC components in *Axd/Axd* embryos leads to increased stress in the surface ectoderm, transmitted via the actomyosin network. This is consistent with alteration of surface ectoderm cell dimensions (midline elongation and increased apical-basal height) in the midline. Moreover, increased recoil in the puncture assay demonstrated an actomysosin-dependent increase in mechanical stress within the *Axd/Axd* surface ectoderm.

We propose that surface ectoderm epithelial integrity is ‘weakened’ by *Grhl2* loss of function and ‘strengthened’ by *Grhl2* over-expression, resulting in failure of spinal neurulation in both cases. Progression of spinal closure depends on the ability of pro-closure forces to overcome anti-closure forces, the latter being revealed by neural fold retraction following laser ablation of the closure point^33^. Increased retraction of the neural folds in *Grhl2* null embryos and resistance to retraction in *Axd/Axd* embryos implies that the anti-closure force principally emanates from the neuroepithelium.

We hypothesise that altered cell–cell junction composition and disruption of the actomyosin cellular network and ‘cable’ renders the *Grhl2* null surface ectoderm less able to accommodate anti-closure forces. This is consistent with increased cell width, suggestive of lateral ‘stretching’ of the surface ectoderm, together with increased tissue recoil after closure point ablation. The closure point is a functionally unique site at which cells extend and interdigitate protrusions and form new cell–cell adhesion. We hypothesise that superimposition of excessive lateral tissue tension at this site impairs propagation of closure.

In contrast to *Grhl2* null embryos, the supra-cellular actomyosin cable is intact at the neural fold margins and midline of *Grhl2* over-expressing embryos. Moreover, the surface ectoderm in this model is able to constrain neural fold separation after laser ablation of the closure point. While increased stress and/or cell–cell adhesion in the *Grhl2* over-expressing surface ectoderm may confer resistance to PNP widening after closure point ablation, this same property may suppress cellular properties required for progression of closure ‘zippering’. For example, we speculate that cells at the leading edge of the neural fold are normally in a ‘less epithelial’ state (on an EMT or epithelial-neuroepithelial spectrum) than cells that are fully integrated within the surface ectoderm. If such an identity shift is normally required for remodelling of cell–cell adhesion at the closure site this could be suppressed by the effects of *Grhl2-*mediated upregulation of epithelial proteins in *Axd/Axd* embryos. Increased abundance of AJCs and surface ectoderm ‘stiffness’ could also disrupt tissue-level integration of cell adhesion and actomyosin contractility (beyond solely the closure site) that is required for the active closure process. In other developmental contexts, including *Drosophila* dorsal closure, epithelial tissue morphogenesis depends on dynamic actomyosin activity, which is integrated with cell–cell adhesion^24,45^. Similarly, during PNP closure actin disassembly and actomyosin turnover are needed in the neuroepithelium^37^. The finding of partial rescue of PNP closure by myosin inhibition in *Axd/Axd* embryos suggests that this is also true in the surface ectoderm.

Overall, the wide PNPs of *Grhl2* null embryos reflect a net increase in widening forces withstood by the zippering point, which cannot be overcome by pro-closure cellular processes. In contrast, the narrow neuropores of *Axd/Axd* embryos suggest failure of closure after lateral tissue tensions have been overcome to elevate and appose the neural folds. These models provide an opportunity to investigate the inter-relationship of rapidly adaptable cell-level tension relevant to behaviours such as migration or partner exchange, which are likely to influence the progression of zippering, from the more incremental and far-reaching tissue-level biomechanical determinants of morphogenetic shape change.

## Methods

### Mice and embryo collection

Animal studies were carried out under regulations of the Animals (Scientific Procedures) Act 1986 of the UK Government, and in accordance with the guidance issued by the Medical Research Council, UK in *Responsibility in the Use of Animals for Medical Research* (July 1993). The *Axd* (*Axial defects*) allele arose as a spontaneous mutation^9^. In this study we denote *Grhl2*^*Axd/Axd*^ as *Axd/Axd* to allow indication of +/+ wild-type littermates, as distinct from *Grhl2*^*+/+*^ littermates of *Grhl2*^*−/−*^.The *Grhl2* gene-trap allele (Grhl2^Gt(AC0205)Wtsi^)^9^ is predicted to be functionally null on the basis of a lack of detectable mRNA expression and is designated *Grhl2*^*−/−*^ for clarity. Both strains of mice were on a BALB/c genetic background. Litters were generated by timed matings in which mice were paired overnight and the day of finding a copulation plug was designated embryonic day 0.5 (E0.5). Embryos were genotyped by PCR of genomic DNA prepared from yolk sacs as previously described^9^. Embryo sex was not determined as spinal NTDs are 90–100% penetrant in both strains^9^.

### PNP measurements, histology and X-gal staining

The PNP length of embryos at E9–10 was measured using an eye-piece graticule. For histological examination, E9.5 embryos were fixed overnight in Bouin’s solution (Sigma), embedded in paraffin-wax at 10 µm and stained with haematoxylin and eosin. For X-gal staining, E9.5 embryos were fixed in ice-cold 0.2% glutaraldehyde and stained at 37 °C overnight as described^9^.

### In situ hybridisation

In situ hybridisation (ISH) used cDNA probes for *Grhl2* and *EpCAM* ^9,46^ and additional probes generated by PCR-based cloning (see Table S4 for primers) into pGEM-T easy vector (Promega). Sense and anti-sense riboprobes were generated using a digoxygenin RNA labelling kit (Roche) and purified on Chroma spin columns (Clontech). After whole-mount ISH^6,47^, embryos were imaged by light stereomicroscope (Leica), embedded in gelatin/albumin and vibratome-sectioned (40 µm). Sections were imaged by light microscopy (Zeiss). Images are representative of three or more embryos at each stage and genotype.

### Western blotting

Snap frozen caudal regions of E9.5 embryos were lysed and sonicated in ice-cold RIPA buffer [1% Nonidet P-40, 50 mM Tris-HCl (pH 8.0), 150 mM NaCl, 0.5 mM EDTA, 1 mM PMSF (Sigma-Aldrich), 1x complete protease inhibitor cocktail (Roche), 1x phosphatase inhibitor cocktail (Sigma-Aldrich)]. Protein concentration was determined using the BCA^TM^ protein assay kit (Pierce). Equal amounts of protein were resolved on 4–12% Bis-Tris protein gels (Nupage, Thermo Fisher Scientific) and electrotransferred onto a polyvinyl difluoride membrane (PVDF) (Millipore). Blots were blocked in 5% non-fat dry milk in 1x TBS-T. Grhl2 (Sigma-Aldrich, HPA004820) or GAPDH (Millipore, MAB374) primary antibodies were applied in the same buffer at a dilution of 1:1000 and 1:10,000, respectively. Secondary antibodies were horseradish peroxidase-conjugated (Dako) and the signal was detected using enhanced chemiluminescence substrates (Pierce). Uncropped blots are found in the Source data file.

### RNA extraction

The caudal region, encompassing the PNP, was isolated from E9.5 embryos at the level of somite 14 and immediately frozen. RNA was extracted (RNeasy mini kit, Qiagen), DNase-treated (Ambion) and processed either for RNA-seq or first strand cDNA synthesis (SuperScript™ VILO™, Thermo Fisher Scientific). In microdissection experiments, the dorsal part and the ventral part of each sample were separated by tungsten needles. RNA extraction was performed using pools of five samples per genotype. In total three biological replicates were processed (i.e., 15 different embryos per sample).

### Quantitative real-time RT-PCR

Quantitative real-time RT-PCR (qRT-PCR) was performed using the iTAQ Universal SYBR Green Supermix assay (Biorad) on a CFX96 system (Biorad) and analysis performed by the 2-^ΔΔCT^ method. Primers for *Grhl2*, *Cldn6*, *Cldn7*, *Vim* and *Zeb2* were as reported^9,48–51^. Additional primers used for qRT-PCR are listed (Supplementary Tables 4 and 5).

### RNA-seq Library preparation and sequencing

Samples were processed using the KAPA mRNA HyperPrep Kit (p/n KK8580) or Illumnina TruSeq Stranded mRNA LT sample preparation kit (p/n RS-122-2101). Briefly, mRNA was isolated from total RNA using Oligo dT, fragmented using chemical fragmentation and primed with random hexamers. Strand-specific first strand cDNA was generated using reverse transcriptase in the presence of Actinomycin D. The second cDNA strand was synthesised using dUTP in place of dTTP and the resultant cDNA was then ‘A-tailed’ at the 3’ end to prevent self-ligation and adapter dimerisation. Truncated adaptors, containing a T overhang were ligated to the A-Tailed cDNA. Successfully ligated cDNA molecules were then enriched with limited cycle PCR (10–14 cycles). Libraries to be multiplexed in the same run were pooled in equimolar quantities, calculated from Qubit and Bioanalyser fragment analysis. Samples were sequenced on a NextSeq 500 (Illumina, San Diego, US) using a 43 bp paired end run resulting in >15million reads per sample.

### RNA-seq analysis

Paired end reads were mapped to the Ensembl mouse transcriptome reference sequence (Mus musculus GRCm38). Mapping and generation of read counts per transcript were performed using Kallisto (10.1038/nbt.3519). R/Bioconductor was used to import the mapped counts data and summarise the transcript-level data at gene level as described^52^ with further analysis using DESeq2 and the SARTools packages^53,54^. Normalisation and differential analysis were carried out according to the DESeq2 model by use of negative binomial generalised linear model. SARTools was used to generate lists of differentially expressed genes. Functional classification of gene lists was performed using Ingenuity Pathway analysis (IPA, https://www.qiagenbioinformatics.com/). Significance of the association of biological functions and canonical pathways were tested by Fisher Exact test. Heat maps were generated using Heatmapper^55^.

### Immunofluorescence

For antibody staining of sections^37^, E9.5 embryos were fixed in 4% PFA overnight or in acetone/methanol (1:1) for 20 min, embedded in 7.5% gelatin/20% sucrose and cryosectioned. Sections were blocked and permeabilized in 10% sheep serum in PBS-Tween (0.1%) and primary antibodies were applied overnight. Sections were washed, incubated with secondary antibody (Alexa Fluor®, Thermo Fisher Scientific, 1:500) at room temperature and counterstained with DAPI. Images were acquired on an inverted LSM710 confocal microscope (Zeiss).

For whole-embryo immunostaining^33^, embryos were blocked and permeabilized in 5% BSA in PBS-Triton X (0.1%) overnight. In the same solution the primary antibody was diluted (1:100 or 1:200) for overnight incubation. Primary antibodies were for E-cadherin (610181, BD Biosciences; 3195 Cell signalling), EpCAM (ab71916, Abcam), Cldn 4 (sc17664, Santa Cruz), ZO-1 (402200, Invitrogen), Vimentin (5741, Cell Signalling), β-catenin (8814, Cell Signalling), N-Cadherin (14215, Cell Signalling), p-MLCII (3671, Cell Signalling), Sox2 (ab92494, Abcam), fibronectin (sc6952, Santa Cruz). After washes in blocking solution (1 h/wash), embryos were incubated with secondary antibody (Alexa Fluor®, Thermo Fisher Scientific, 1:500 in blocking solution). After further washes (1 h/wash), the embryos were either stained with phalloidin (Alexa Fluor™ 568 or 647, Thermo Fisher Scientific) or counterstained in DAPI and processed for imaging. For Cldn4, N-cadherin, Vimentin and Sox2 immunostaining, embryos were fixed with 4% PFA overnight and antigen retrieval was performed using citric acid buffer pH 6.0 at 90 °C for 1 h, followed by cooling to room temperature prior to blocking/permeabilization. Images were acquired on a Zeiss Examiner LSM880 confocal microscope using a 20 × /NA1.0 Plan Apochromat dipping objective. High-resolution images were obtained with Airyscan in SR mode with optimal pixel size and Z-step. Images were processed with Zen2.3 software and visualised as maximum projections in ImageJ/Fiji. Analysis of whole-mount immunofluorescent images was performed using an ImageJ/Fiji in-house macro (https://bit.ly/2CGMI4Q) in order to visualise the surface ectoderm layer^56^.

### Surface ectoderm segmentation

Surface ectoderm cell segmentation for cell shape analysis was done using Tissue Analyser in ImageJ/Fiji^57^ based on the maximum projection of ZO-1 stained whole-mount immunofluorescent images. The outline of cells within a defined region was traced using Fiji. The length:width ratio of these cells was calculated using measurements generated by the Fiji Plugin programme Ellipse. For the segmentation of surface ectoderm cells in the clusters, ImageJ/Fiji was used in Airyscan images. Three-dimensional (3D) viewer plugin was used for the 3D display.

For measuring apical-basal thickness of the surface ectoderm, cells were manually segmented using Seedwater segmenter software^58^ in cross-sectional z-stacks of E-cadherin immunostained transverse sections. Tissue curvature was corrected by tracing a line along the apical surface of the cell layer in each embryo and straightening the images using the ‘straighten’ function in Fiji. Surface ectoderm thickness was quantified at repeated intervals moving laterally from the midline. Symmetry around the zippering point was assumed and the left and right side of each embryo were averaged. Thickness was compared between genotypes using a mixed models procedure in IBM SPSS Statistics v.22, with genotype and position lateral to the zippering point as fixed factors, and accounting for repeated measurements from each embryo. Genotype by position interaction was not significant and was removed from the final model. A post-hoc Bonferroni correction was applied to identify positions at which the genotypes diverged significantly.

### Scanning and transmission electron microscopy

For SEM, embryos were prepared by overnight fixation in 2% glutaraldehyde, 2% paraformaldehyde in 0.1 M phosphate buffer, pH 7.4, at 4 °C, post-fixed in 1% OsO4/1.5% K4Fe(CN)6 in 0.1 M phosphate buffer at 3 °C for 1.5 h and rinsed in 0.1 M phosphate buffer, followed by distilled water. Samples were dehydrated through an ethanol series, washed in acetone and critical point dried using CO_2_, then mounted on aluminium stubs using sticky carbon taps. Mounted samples were coated with alayer of Au/Pd (~2 nm thick) using a Gatan ion beam coater and imaged with a JEOL 7401 FEGSEM. Double-blind scoring of the protrusions was performed using SEM images of the closure point^3^. Cell shape analysis was performed on SEM images of the midine region. Analysis of the angle between the neural folds was performed using the angle tool in ImageJ/Fiji and by measuring the diameter of a circle, drawn with best fit at the closure point (see Fig. 1 and Supplementary Fig. 1).

For transmission electron microscopy, embryos were fixed with 2% paraformaldehyde, 2% glutaraldehyde, 2% sucrose in 0.1 M cacodylate buffer pH 7.3 and post-fixed in 1% OSO4/ 0.1 M Cacodylate buffer pH 7.3 at 3 °C for 90 min. After washing in 0.1 M Cacodylate buffer pH 7.4, en bloc staining was performed with 0.5% uranyl acetate at 3 °C for 30 min. Specimens were dehydrated in a graded ethanol-water series and infiltrated with Agar 100 resin mix overnight, then hardened at 60 °C for 24 h. One micrometer sections were stained with 1% toluidine blue for light microscopy. Ultra-thin sections were cut at 70–80 nm using a diamond knife on a Reichert Ultracut microtome. Sections were collected on carbon/formvar slot grids and then stained with lead citrate. Sections were viewed in a Joel 1010 transition electron microscope and images recorded using a Gatan Orius camera.

### Surface ectoderm puncture experiments

After dissection of E9.5 embryos, the dorsal surface ectoderm rostral to the PNP was punctured by a microsurgical pin (Ethicon) followed by immediate fixation. Yolk sacs were retained for genotyping. Punctures were also performed after embryo culture (see below) in the presence of either blebbistatin (50 µM; Calbiochem) or vehicle (DMSO 1:1000) for 8 h. Embryos were stained with CellMask (Thermo Fisher Scientific) and imaged using LSM880 confocal microscope. The major/minor axis of the puncture was measured using ImageJ/Fiji.

### Laser ablation of the PNP closure point

Embryos at E9.5 were dissected, positioned in agarose dishes in dissection medium (maintained at 37 °C) and held in place using microsurgical needles with the PNP facing upwards. Laser ablations of 300–500 μm were performed along the most recently closed neural tube^33^, on a Zeiss Examiner LSM880 confocal microscope using a 20 × /NA1.0 Plan Apochromat dipping objective and a SpectraPhysics Mai Tai eHP DeepSee multiphoton laser. Images of the embryos before and after ablation were captured using the same settings. Resulting z-stacks were reoriented and resliced in ImageJ/Fiji.

### Embryo culture

Litters were dissected, leaving the yolk sac and ectoplacental cone intact. For measurement of closure progression, a window was opened in the yolk sac over the PNP using fine scissors, and the location of the closure point was marked by injection of CM-DiI (C7001, Cell Tracker, Molecular Probes). A mouth-controlled micropipette was inserted through the amnion and a small amount of DiI was expelled beneath the surface ectoderm, lateral to and level with the closure point. Embryos were cultured in rat serum containing either vehicle (DMSO, 1:1000) or blebbistatin (50 µM), gassed with 20% O_2_, 5% CO_2_ and 75% N_2_, and incubated with rolling at 37 °C for 8 h^59^. After culture, the distance between the DiI mark and the caudal end of the embryo and the PNP length were measured using an eye-piece graticule. Embryos were imaged with an LSM880 confocal microscope using the reflection settings in order to record the PNP morphology (see also Fig. 9c, d).

### Statistical analysis

Statistical analysis was performed using IBM SPSS Statistics 22 and Sigmastat version 3.5 (Systat Software). For box and whisker plots (Figs. 1 and 8) centre line, median; box limits, upper and lower quartiles; whiskers, range.

### Reporting summary

Further information on research design is available in the Nature Research Reporting Summary linked to this article.

## Supplementary information

Supplementary Information

Reporting Summary

Source Data file

## Data Availability

The authors declare that all data supporting the findings of this study are available within the article and its supplementary information files or from the corresponding author upon reasonable request. RNA-seq data have been deposited at NCBI Sequence Read Archive BioProject under accession code: PRJNA534430. Source data for Figs. 1–3 and 6–9 and Supplementary Figures are provided as a Source Data file.
