# Supplementary material for: Spinal neural tube closure depends on regulation of surface ectoderm identity and biomechanics by Grhl2
Source: Nat Commun. 2019 Jun 6;10:2487. doi: 10.1038/s41467-019-10164-6 (PMC6554357; doi:10.1038/s41467-019-10164-6)
Supplement: Supplementary file 1 — Supplementary Information [file 41467_2019_10164_MOESM1_ESM.pdf]

Supplementary Information

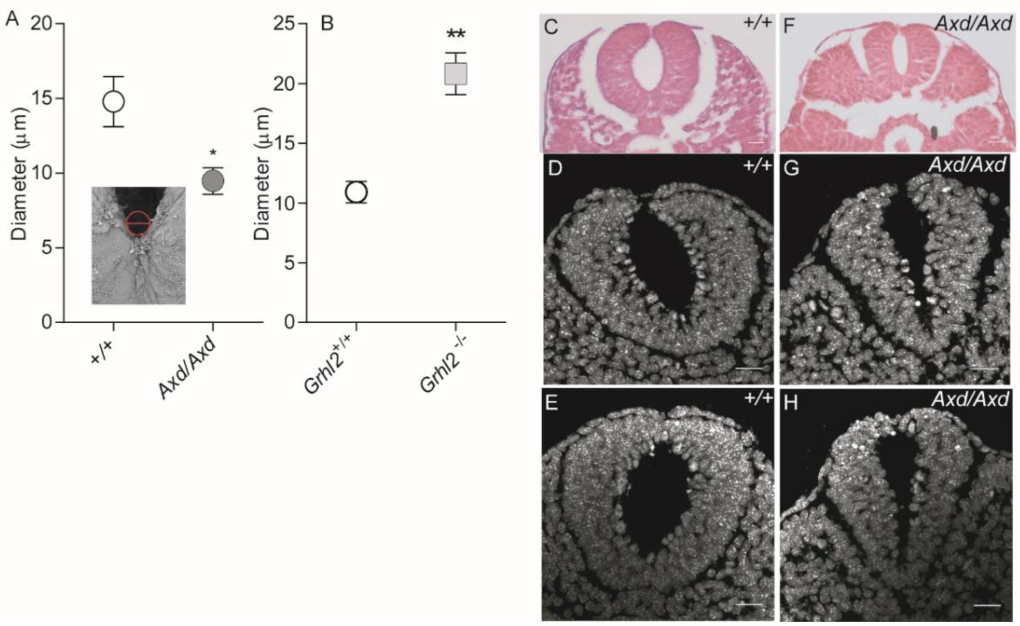

Supplementary Figure 1. Closure point and neural fold morphology of *Axd* mutants at E9-9.5 (Related to Figure 1) (A-B) The angle between the neural folds was evaluated by determination of the diameter of a circle drawn at the closure point on a dorsal view (a larger diameter corresponds to increased spacing between the neural folds). The neural fold spacing at the closure point is (A) significantly smaller in *Axd/Axd* mutant embryos than in wildtype littermates (n ≥ 5 per genotype; E9.5), whereas (B) the closure site in *Grhl2*$^{-/-}$ is wider than among *Grhl2*$^{+/+}$ embryos (n = 4 per genotype; E9.5). Data indicate mean ± SEM (*p<0.05, t-test). (C-H) Transverse sections at the closure site (C-H) show that *Axd/Axd* and +/+ embryos lack obvious DLHPs at E9 (C, F; 13-14 somite stage). At E9.5 (D-H, 19-20 somite stage) the neural folds of the *Axd* mutants (G and H) appear to bend at a more dorsal position than in wildtype controls (D-E). Scale bars represent 25µm (C, F) and 20µm (D, E, G, H). Images are representative of at least 3 different embryos per genotype. Source data are provided as a Source Data file.

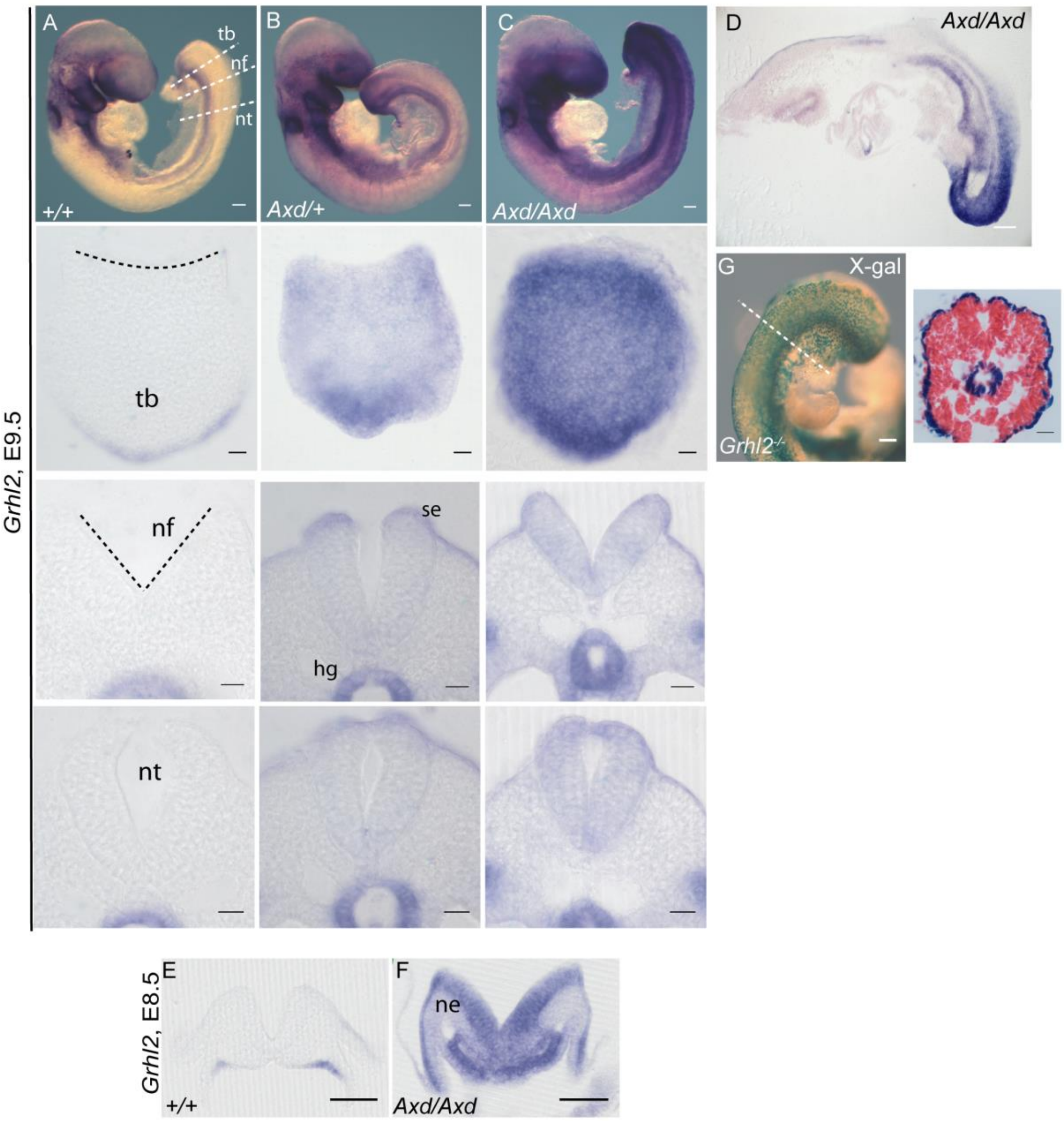

Supplementary Figure 2. Over-expression of *Grhl2* in *Axd/Axd* embryos (Related to Figure 2) (A-C) Whole mount *in situ* hybridisation for *Grhl2*. A-C are replicated from Fig. 2 to show levels of sections at the tail bud (tb), open neural folds (nf) and recently closed neural tube (nt). *Grhl2* is expressed in the surface ectoderm, hindgut and ventral ectodermal ridge in wildtype embryos (A) and is up-regulated in the tail bud of *Axd/+* (B) and *Axd/Axd* (C) embryos. (D) Sagittal section through the caudal region of an *Axd* mutant following WMISH for *Grhl2*. (E-F) Transverse section through the PNP at E8.5 (WMISH in Fig. 2E-F). (G) X-gal staining of a caudal region and a transverse section at the level of the PNP closure point of a *Grh2*$^{-/-}$ embryo, shows expression of β-galactosidase from the *Grhl2* locus and confirms expression in the surface ectoderm and hindgut. Scale bars: 100 μm in whole mount and 25 μm in section. Images are representative of at least 3 different embryos per genotype.

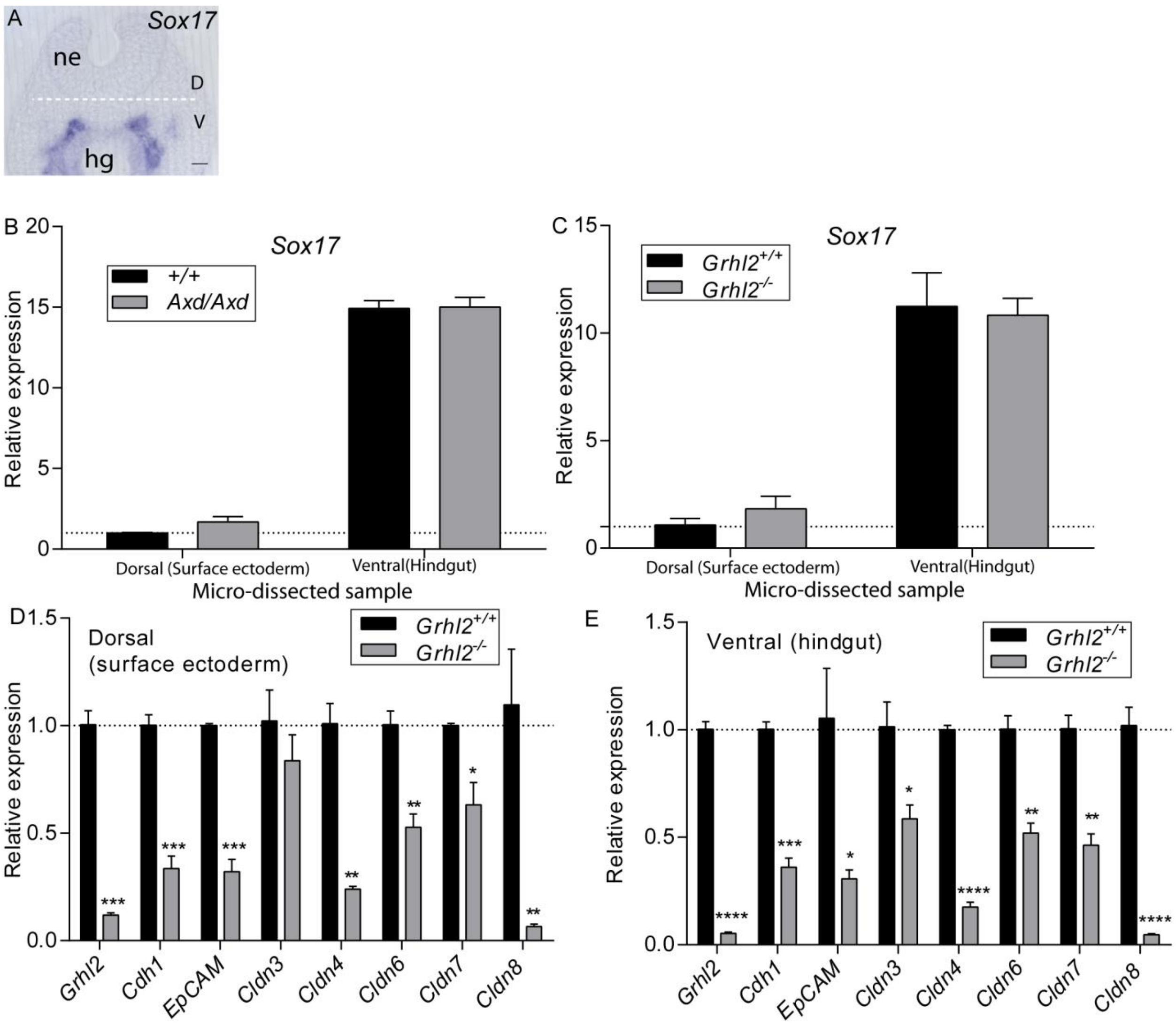

Supplementary Figure 3. Validation of microdissection of dorsal and ventral tissue and qRT-PCR analysis of *Grhl2*$^{-/-}$ samples (Related to Figure 3) (A) A transverse section of the PNP region of a wildtype embryo following whole mount *in situ* hybridisation for *Sox17.* The dotted line shows the position of the microdissection (ne, neuroepithelium; hg, hindgut). (B-C) *Sox17* expression, determined by qRT-PCR of micro-dissected samples, was higher in the ventral (hindgut-containing; *Sox17*-positive) tissue than in dorsal (surface ectoderm-containing) samples as predicted, and did not vary between mutant and wildtype embryos. (D-E) qRT-PCR analysis for the dissected dorsal (D) and (E) ventral samples from *Grhl2*$^{-/-}$ and *Grhl2*$^{+/+}$ samples (n= 5 embryos per sample, with 3 samples per genotype); Values indicate mean ±SEM ****p<0.0001, ***p<0.001, **p<0.01, *p<0.05 (Student's t-test). Source data are provided as a Source Data file.

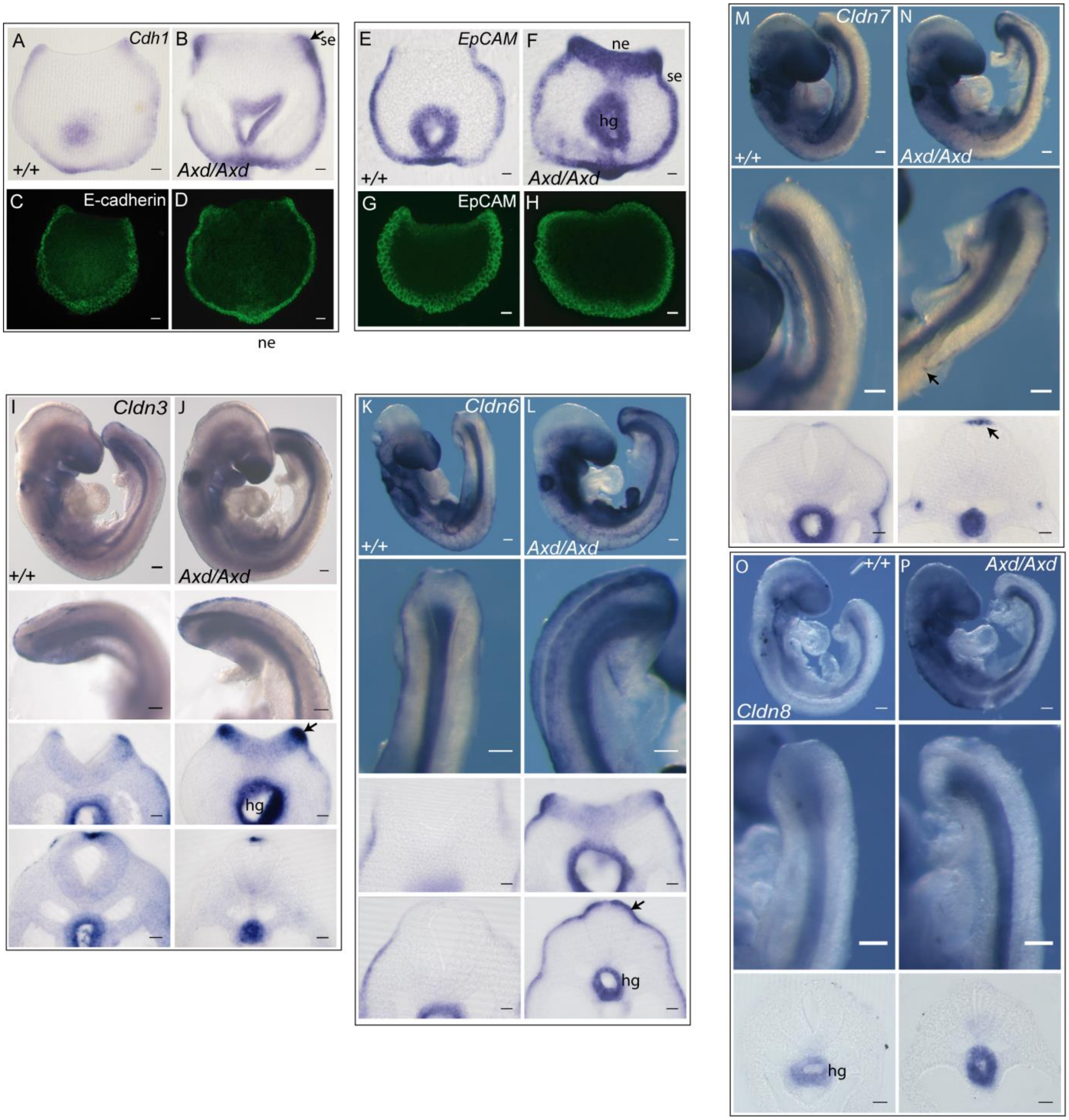

Supplementary Figure 4. Elevated expression of *Cdh1*, *EpCAM* and *Cldn3, 6, 7* and *8* in *Axd* mutant embryos (Related to Figure 3) WMISH and transverse sections for (A-B) *Cdh1*, (E-F) *EpCAM*, (I-J) *Cldn3*, (K-L) *Cldn6* and (M-N) *Cldn7* show more intense staining in *Axd/Axd* than in +/+ embryos in the surface ectoderm (black arrows). (O-P) *Cldn8* is up-regulated in *Axd/Axd* embryos in the hindgut only. (C-D, G-H) Immunostaining shows that E-cadherin (C-D) and EpCAM (G-H) localisation in the surface ectoderm is not altered in *Axd* mutants in the tail bud. Scale bars: 100 µm in whole embryos; 25µm transverse sections (se, surface ectoderm; ne, neuroepithelium; hg, hindgut).

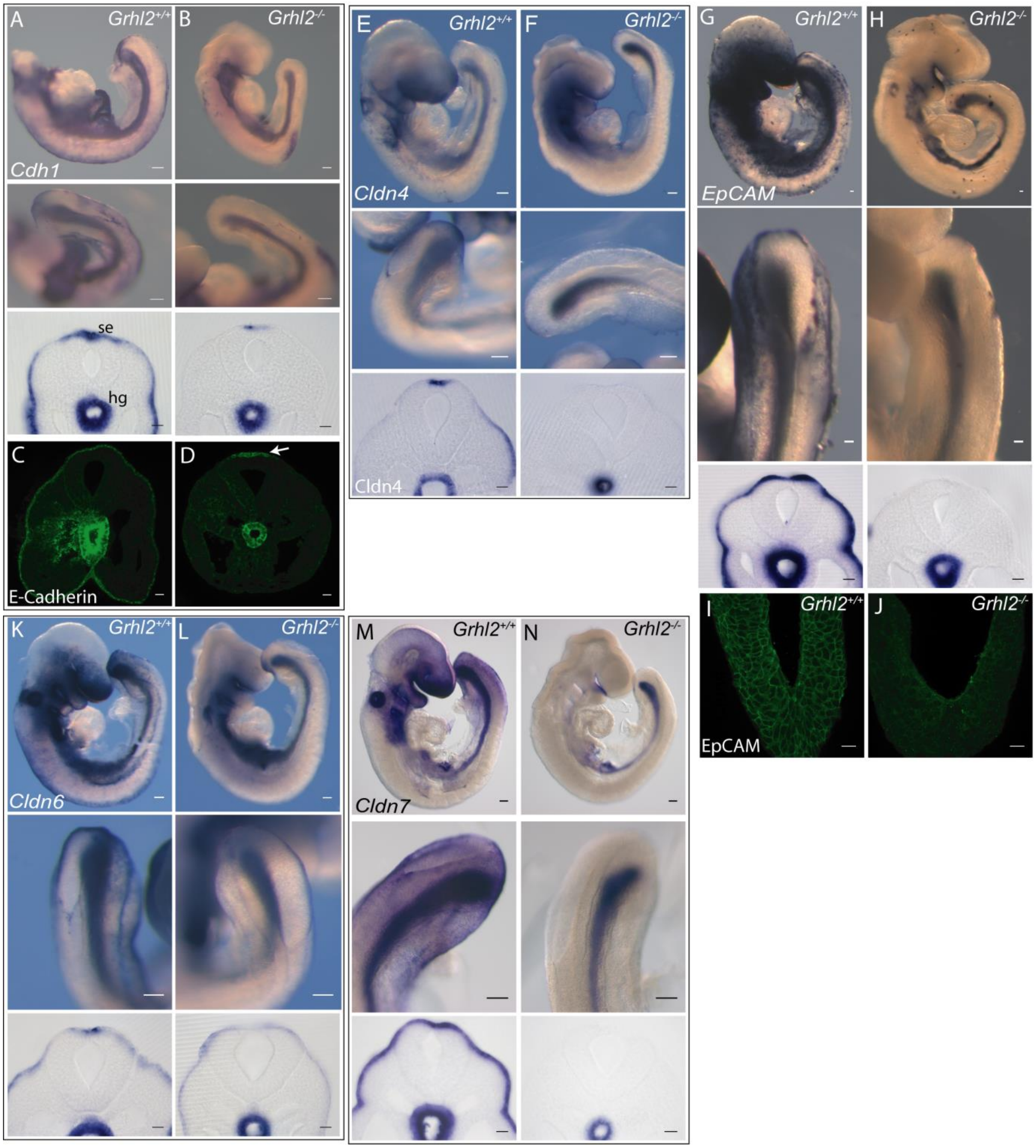

Supplementary Figure 5. Down-regulation of epithelial markers in *Grhl2* null embryos (Related to Figure 4) WMISH shows lower expression of (A-B) *Cdh1*, (E-F) *Cldn4*, (G-H) *EpCAM*, (K-L) *Cldn6* and (M-N) *Cldn7* in *Grhl2*$^{-/-}$ embryos than in *Grhl2*$^{+/+}$ embryos at E9.5 (panels show higher magnification views of the caudal region). Diminished expression in the surface ectoderm at the level of the closure point was confirmed in the transverse sections. (C-D) A transverse section of the recently closed neural tube shows lower abundance of E-cadherin (white arrow indicates residual E-cadherin) in *Grhl2*$^{-/-}$. (I-J) Dorsal views of whole mount immunostaining for EpCAM. Scale bars: 100 µm in whole mount images and 25 µm in sections.

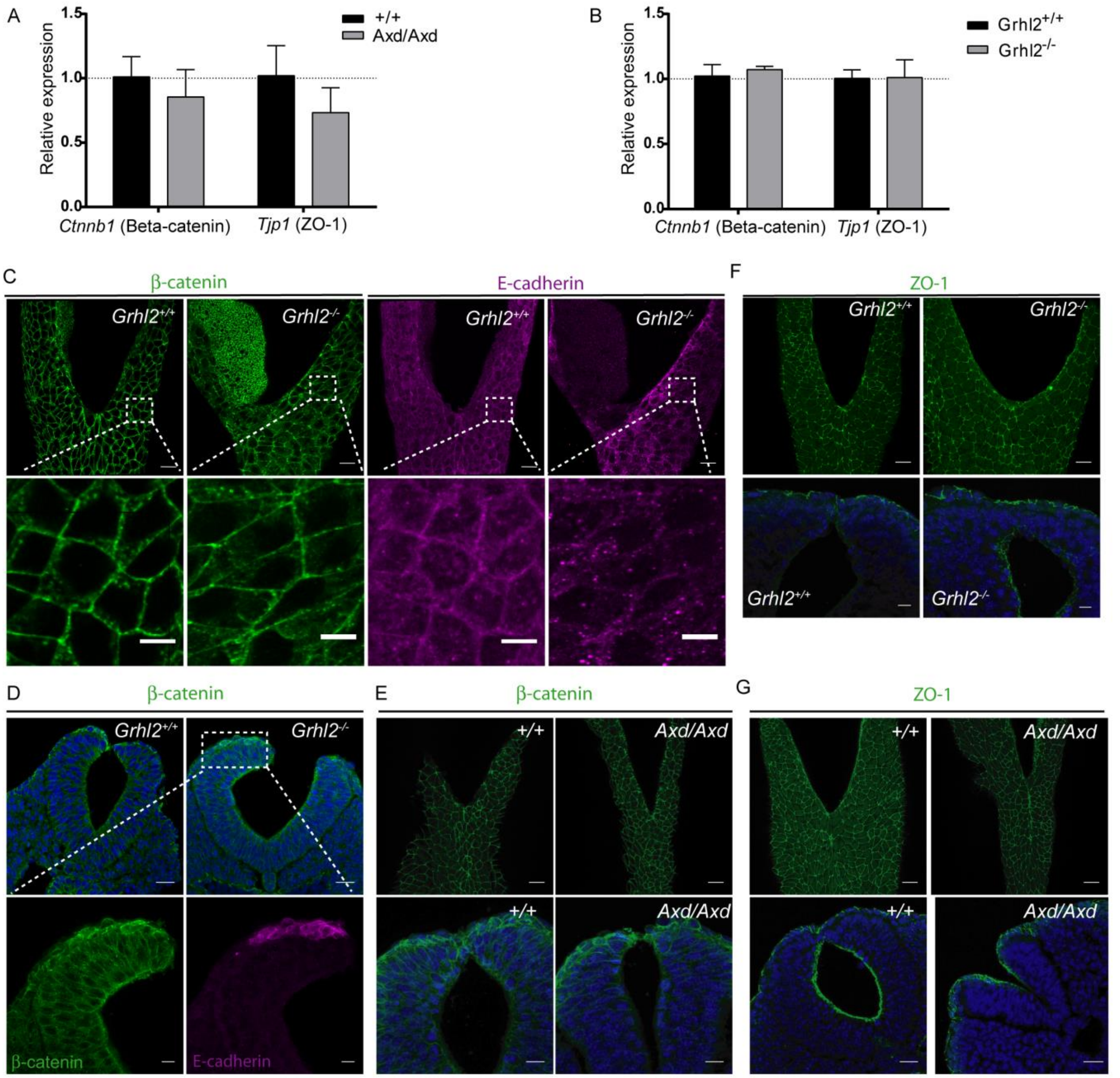

Supplementary Figure 6: Beta-catenin and ZO-1 expression in *Grhl2* mutants (Related to Figure 4) (A-B) Expression of *Ctnnb1* and *Tjp1* was determined by qRT-PCR of samples derived from the caudal regions (as Fig. 3A) in embryos at E9.5 (n = 3-6 per group; 17-19 somite stage). Expression did not differ from stage-matched wildtype controls in (A) *Grhl2*$^{-/-}$ or (B) *Axd/Axd* embryos. (C) Surface-subtracted images of dorsal view of PNP regions and (D) transverse sections of *Grhl2*$^{+/+}$ and *Grhl2*$^{-/-}$ embryos at E9.5 (examples have 20-21 somites) following immunostaining for β-catenin (green) and E-cadherin (magenta). β-catenin localised at cell boundaries in the surface ectoderm, even in regions of low E-cadherin abundance in *Grhl2*$^{-/-}$ embryos (magnified areas in C and D). Scale bars: 25 μm for whole mount images and sections and 10 μm for magnified areas (in C and D). (E) Immunostaining of whole-mount (top row; dorsal view of PNP) and sections (bottom row) for β-catenin (as in C) of *Axd/Axd* and +/+ (examples have 17-18 somites). (F-G) Whole mount (top panels) and transverse sections (bottom panels) of ZO-1 immunostaining in stage-matched *Grhl2*$^{-/-}$ (F) and *Axd/Axd* (G) embryos at E9.5. Scale bars in E-G represent 25 μm on whole mount images, 10 μm on sections.

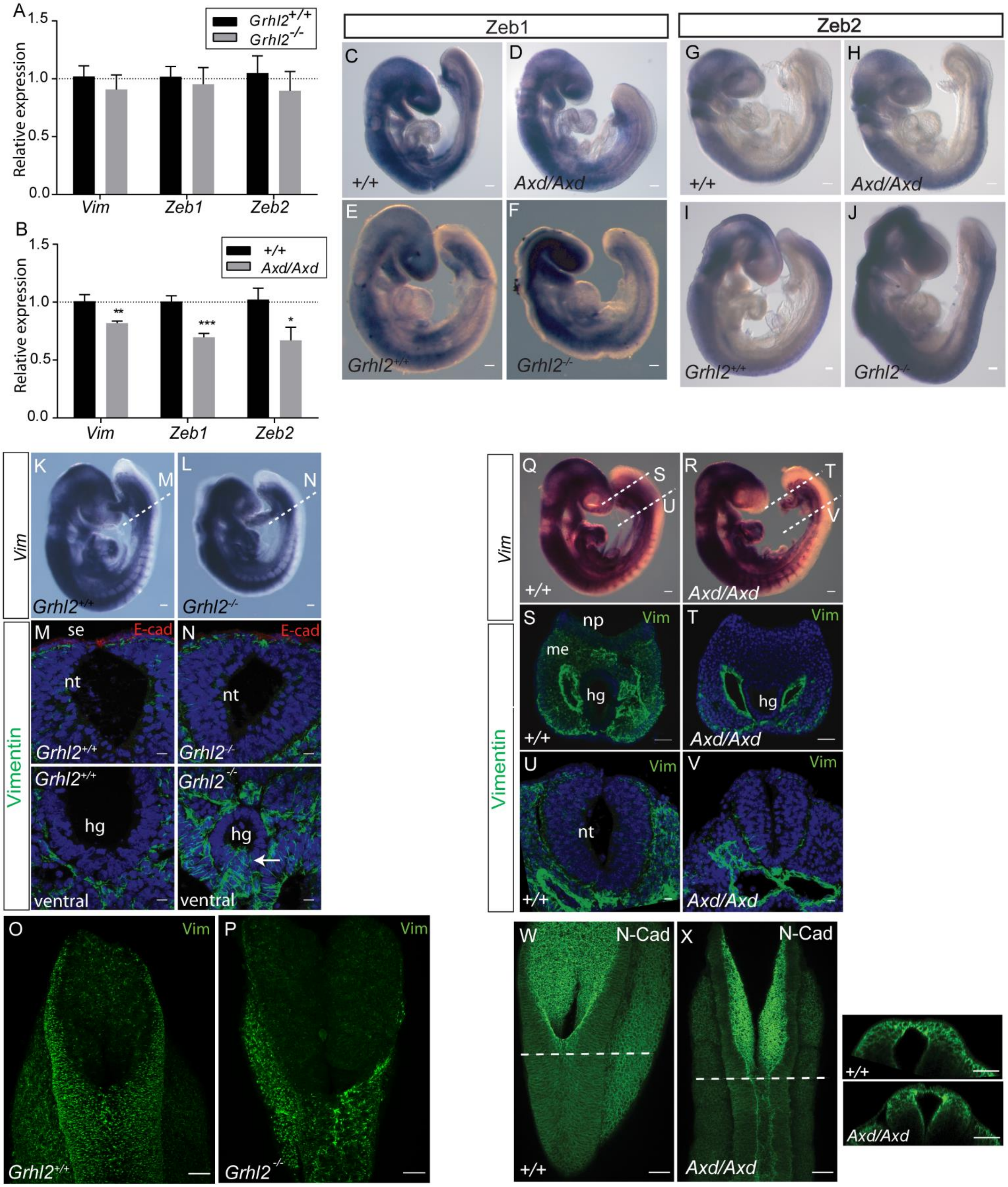

Supplementary Figure 7.Expression of vimentin, Zeb-1 and Zeb-2 in *Grhl2* mutant embryos (Related to Figure 4) (A-B) mRNA abundance of *Vim*, *Zeb-1* and *Zeb-2* quantified by qRT-PCR in isolated caudal regions (as in Fig. 3A) of (A) *Grhl2*$^{-/-}$ and (B) *Axd/Axd* embryos at E9.5 (somite stages 17-19). Data

represent mean ± SEM, n = 6 per genotype (***p<0.001, **p<0.01, *p<0.05; t-test). WMISH for *Zeb1* (C-F), *Zeb2* (G-J) and *vimentin* (K-L, Q-R) in *Axd/Axd* and *Grhl2*$^{-/-}$ embryos and wildtype littermates. (M-N) Immunostaining for vimentin (green) at E9.5 is found subjacent to the E-cadherin-positive surface ectoderm (red) at the PNP closure point and in the ventral hindgut (hg) in *Grhl2* null embryos (arrow in ventral panel, N). (O-P) Whole mount immunostaining for vimentin in the spinal region at E9.5 (positive staining corresponds to the basal neuroepithelium). (S-V) In *Axd/Axd* mutants, vimentin (green) is less abundant in the neural tube (nt) and presomitic mesoderm (me) in the caudal PNP region (S-T) and adjacent to the PNP closure point (U-V). Sections through the spinal region are at the level shown by dashed lines in K-L and Q-R. (W-X) Immunostaining for N-cadherin; dashed lines indicate the level of sections. Scale bars: 10 µm in M-N, U-V; 25 µm in S-T, 50 µm in O-P, W-X and 100 µm in whole mount images. Source data are provided as a Source Data file.

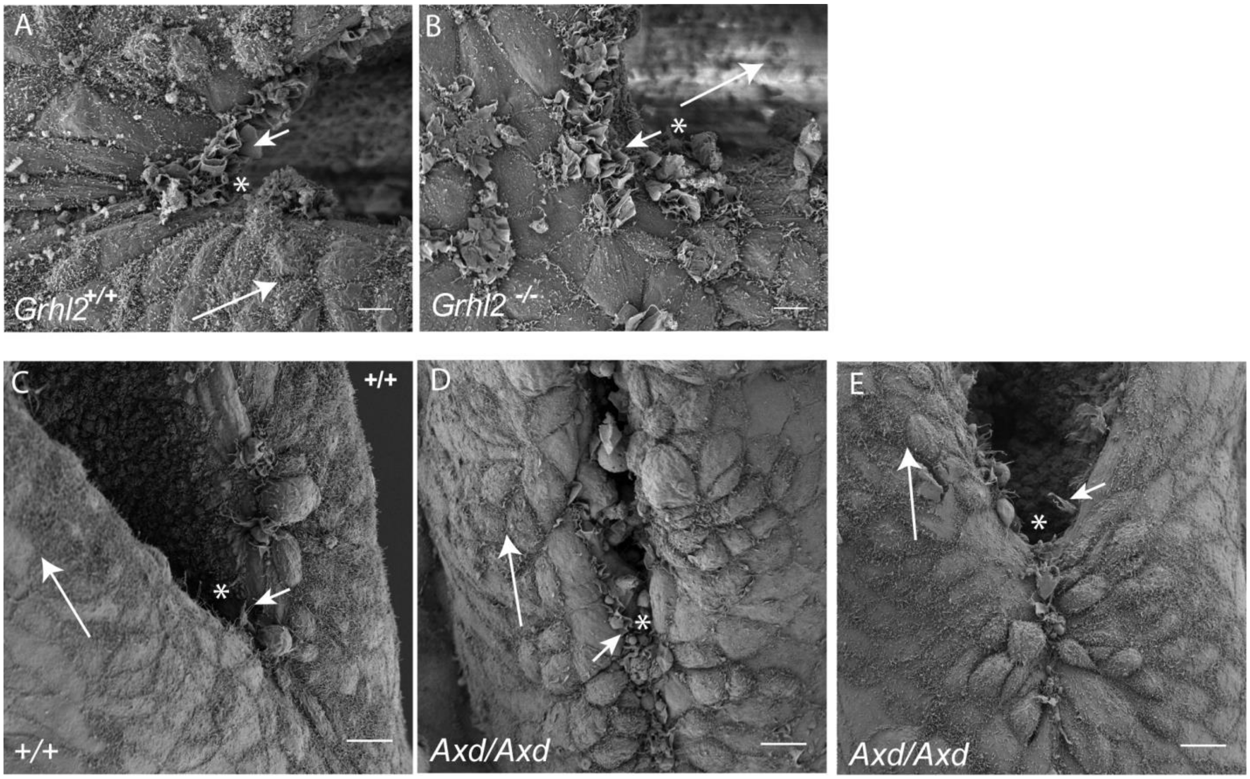

Supplementary Figure 8: Characteristic cellular protrusions are present at the medial edge of the neural folds in wildtype and Grhl2 null or over-expressing (*Axd*) embryos (Related to Figure 5). Representative scanning electron micrographs showing the presence of protrusions (short arrows) at the PNP closure points (indicated by *) of wildtype (A, C) *Grhl2*$^{-/-}$ (B) and *Axd/Axd* (D-E) embryos at E9.5 (examples have 16-20 somites). Long arrows indicate direction of rostral-caudal axis. Membrane protrusions are present at the surface ectoderm/ neuroepithelium boundary of the neural folds in all genotypes and include predominantly ruffles (white arrows) with filopodia also present. Total embryos analysed: n $\geq$ 5 per genotype.

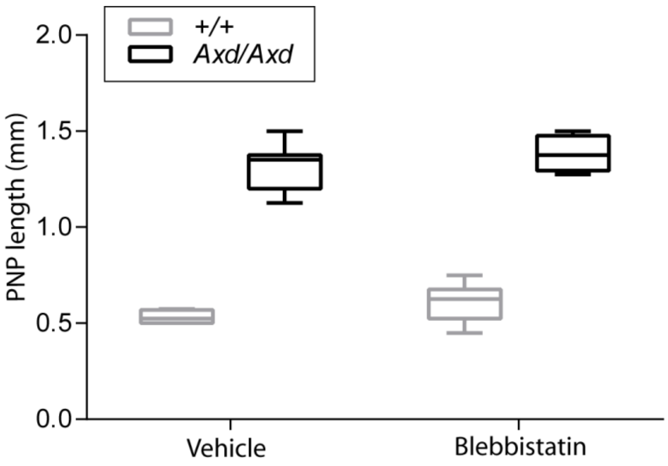

Supplementary Figure 9. Posterior neuropore length embryos after culture in the presence of blebbistatin (Related to Figure 8). Posterior neuropore (PNP) lengths were measured among embryos cultured for 8 hours in the presence of blebbistatin (50µM) or vehicle only (effective dose is lower in this experiment than in Fig. 9 as embryos were cultured within the intact yolk sac). All embryos were at E9.5 (19-23 somite stage at the end of culture period). PNP length did not differ between treatment groups within each genotype. N $\geq$ 4 per genotype per treatment. Source data are provided as a Source Data file.

Number of somites	*+/+*	*Axd/+*	*Axd/Axd*	*Grhl2*$^{+/+}$	*Grhl2*$^{+/-}$	*Grhl2*$^{-/-}$
**10-12**	7	18	6	6	9	4
**13-15**	10	38	12	16	29	11
**16-18**	21	43	19	31	43	44
**19-21**	12	22	7	26	55	33
**Total**	50	121	44	79	136	92

Supplementary Table 1: Number of embryos for which PNP length measurements were made at E8.5-E9.5 (plotted in Fig. 1A-B).

**Canonical pathways - *Axd/Axd***		
**Name**	**p-value**	**Overlap**
Sertoli Cell-Sertoli Cell Junction Signaling	1.79E-09	6.2 % 11/178
Tight Junction Signaling	2.22E-06	4.8 % 8/167
ErbB Signaling	1.44E-04	5.1 % 5/98
Signaling by Rho Family GTPases	2.71E-04	2.8 % 7/247
Agrin Interactions at Neuromuscular Junction	4.23E-04	5.8 % 4/69

**Diseases and Disorders – *Axd/Axd***		
**Name**	**p-value**	**#Molecules**
Cancer	3.71E-03 - 1.09E-09	102
Organismal Injury and Abnormalities	3.71E-03 - 1.09E-09	103
Reproductive System Disease	3.71E-03 - 1.09E-09	60
Skeletal and Muscular Disorders	3.71E-03 - 7.02E-06	20
Tumor Morphology	2.93E-03 - 1.18E-05	12

**Canonical pathways -Grhl2$^{-/-}$**		
**Name**	**p-value**	**Overlap**
Sertoli Cell-Sertoli Cell Junction Signaling	2.00E-06	4.5 % 8/178
Regulation of the Epithelial-Mesenchymal Transition Pathway	2.73E-04	3.2 % 6/189
Tight Junction Signaling	1.17E-03	3.0 % 5/167
p53 Signaling	1.88E-03	3.6 % 4/111
Molecular Mechanisms of Cancer	1.95E-03	1.9 % 7/374

**Diseases and Disorders-Grhl2**		
**Name**	**p-value**	**#Molecules**
Cancer	9.39E-03 - 5.51E-07	96
Organismal Injury and Abnormalities	9.39E-03 - 5.51E-07	98
Reproductive System Disease	9.39E-03 - 5.51E-07	47
Gastrointestinal Disease	9.39E-03 - 2.30E-05	85
Endocrine System Disorders	7.47E-03 - 3.15E-05	21

Supplementary Table 2: Ingenuity Pathway Analysis (IPA) of the differentially expressed genes in *Axd/Axd* and *Grhl2*$^{-/-}$ RNA-seq sets (Related to Figure 3)

	+/+	*Axd/Axd*	*Grhl2*$^{+/+}$	*Grhl2*$^{-/-}$
**Number of embryos**	5	5	3	3
**No. midline cells analysed**	33	48	19	22
**No. lateral cells analysed**	82	102	67	43

Supplementary Table 3: Number of cells analysed for cell shape analysis by scanning electron microscopy (Figure 6).

Table S4: Primers used for the cloning of cDNA probes.

Gene	*Forward primer 5'-3'*	*Reverse primer 5'-3'*
Cdh1	GAGAAGACGCTGAGCATGTG	GTAGTCCTGGTCCTGATCCG
Cldn3	CGGTCAGATGCAGTGCAAAA	CAGACGTAGTCCTTGCGGTC
Cldn4	GGCGTCTATGGGACTACAGG	GGTTGTAGAAGTCGCGGATG
Cldn6	TCTGCAAATCTTGGGGATCGT	GAGCAGGCGCAGCATAGTAG
Cldn7	GCCATCCCTCAGTGGCAGAT	TACGCAGCTTTGCTTTCACTG
Cldn8	ATGGCAACCTACGCTCTTCAAA	CTACACATACTGACTTTTGGA
Zeb1	GCGGCGCAATAACGTTACAA	TGACGTTCAAGCTGGGTTCT
Vim	AAGACTCGGTGGACTTCTCG	CCGTTCAAGGTCAAGACGTG
Sox17	GGATACGCCAGTGACGACC	CATGTGCGGAGACATCAGCG

Primer pairs were used to amplify cDNA fragments which were cloned into pGEM-T for production of anti-sense probes for *in situ* hybridisation. Additional probes were for *Grhl2* [1], *Epcam* [2] and *Zeb2*[3].

Supplementary Table 5: List of primers used for qRT-PCR.

Gene	*Fwd primer 5'-3'*	*Rv primer 5'-3'*
Cdh1 (Ecadherin)	TGCCATCCTCGGAATCCTTG	TTTGACCACCGTTCTCCTCC
EpCAM	AAGCCCGAAGGGGCGATCCA	GTAGGTCCTCACGCGCTCGG
Cldn3	TTTTCCTGTTGGCGGCTCTG	GTACAACCCAGCTCCCAT
Cldn4	GGCGTCTATGGGACTACAGG	AGCGCACAACTCAGGATGAT
Cldn8	TTGCTGACAGCCGGAATCAT	GAATTGGCAACCCAGCTGAC
Zeb1	GCAGTCCCACACGCCCACAG	ACGGCTTGCACCACACCCTG
Sox17	AGCCATTTCCTCCGTGGTGT	AACACTGCTTCTGGCCCTCAG
GAPDH	CATACCAGGAAATGAGCTTG	ATGACATCAAGAAGGTGGTG
Ctnnb1 (β- catenin)	GCACCCACCATCCCACTGGC	CTGCTGCTGCGTTCCACCCA
Tjp1 (ZO-1)	GCTAAGAGCACAGCAATG	CCACCAGAGATTGCAATTCCA

Additional published primer sequences were for *Grhl2* [1], *Cldn6* [4], *Cldn7* [5], *Vimentin* [6], *Zeb2* [7].

Supplementary References

1. Brouns,M.R. et al. Over-expression of Grhl2 causes spina bifida in the Axial defects mutant mouse. Hum. Mol. Genet. **20**, 1536-1546 (2011).
2. Nagao, K. et al. Abnormal Placental Development and Early Embryonic Lethality in EpCAM-Null Mice. PLoS One. **4**, e8543 (2009).
3. Weng, Q. et al. Dual-model modulation of Smad signaling by Smad-interacting protein Sip1 is required for myelination in the central nervous system. Neuron **73**, 713-728 (2012).
4. Luo, Y. et al. Differential expression of claudins in retinas during normal development and the angiogenesis of oxygen-induced retinopathy. Invest Ophthalmol Vis Sci. **52**, 7556-64 (2011)
5. Blackman et. al, 2005 Claudin 7 expression and localization in the normal murine mammary gland and murine mammary tumors Breast Cancer Res. **7**, R248–R255 (2005).
6. Yates, B. et al., 2007 Promoter-independent regulation of vimentin expression in mammary epithelial cells by val12*ras* and TGFβ Exp Cell Res. **313**, 3718–3728 (2007).
7. Xiang et al., 2012 Grhl2 determines the epithelial phenotype of breast cancers and promotes tumor progression. PLoS One. 2012;**7**, e50781 (2012).
