# Supplementary material for: Spinal neural tube closure depends on regulation of surface ectoderm identity and biomechanics by Grhl2
Source: Nat Commun. 2019 Jun 6;10:2487. doi: 10.1038/s41467-019-10164-6 (PMC6554357; doi:10.1038/s41467-019-10164-6)
Supplement: Supplementary file 2 — Reporting Summary [file 41467_2019_10164_MOESM2_ESM.pdf]

nature research

Corresponding author(s): N Greene

Last updated by author(s): Apr 5, 2019

Reporting Summary

Nature Research wishes to improve the reproducibility of the work that we publish. This form provides structure for consistency and transparency in reporting. For further information on Nature Research policies, see Authors & Referees and the Editorial Policy Checklist.

Statistics

For all statistical analyses, confirm that the following items are present in the figure legend, table legend, main text, or Methods section.

n/a	Confirmed	
☐	☒	The exact sample size (n) for each experimental group/condition, given as a discrete number and unit of measurement
☐	☒	A statement on whether measurements were taken from distinct samples or whether the same sample was measured repeatedly
☐	☒	The statistical test(s) used AND whether they are one- or two-sided<br>*Only common tests should be described solely by name; describe more complex techniques in the Methods section.*
☐	☒	A description of all covariates tested
☐	☒	A description of any assumptions or corrections, such as tests of normality and adjustment for multiple comparisons
☐	☒	A full description of the statistical parameters including central tendency (e.g. means) or other basic estimates (e.g. regression coefficient) AND variation (e.g. standard deviation) or associated estimates of uncertainty (e.g. confidence intervals)
☐	☒	For null hypothesis testing, the test statistic (e.g. F, t, r) with confidence intervals, effect sizes, degrees of freedom and P value noted<br>*Give P values as exact values whenever suitable.*
☒	☐	For Bayesian analysis, information on the choice of priors and Markov chain Monte Carlo settings
☒	☐	For hierarchical and complex designs, identification of the appropriate level for tests and full reporting of outcomes
☒	☐	Estimates of effect sizes (e.g. Cohen's d, Pearson's r), indicating how they were calculated

Our web collection on statistics for biologists contains articles on many of the points above.

Software and code

Policy information about availability of computer code

Data collection	For RNA-Seq, mapping and generation of read counts per transcript were performed using Kallisto (doi:10.1038/nbt.3519).<br>Imunofluorescence images were processed with Zen2.3 software (Zeiss) and visualised as maximum projections in ImageJ/Fiji. Analysis of whole-mount immunofluorescent images was performed using an ImageJ/Fiji in-house macro (https://bit.ly/2CGMI4Q) in order to visualise the surface ectoderm layer.
Data analysis	Surface ectoderm cell segmentation for cell shape analysis was done using Tissue Analyser in ImageJ/Fiji (Aigouy,B., Umetsu,D., & Eaton,S. Segmentation and Quantitative Analysis of Epithelial Tissues. Methods Mol. Biol. 1478, 227-239, 2016).<br>For measuring apical-basal thickness of the surface ectoderm, cells were manually segmented using Seedwater segmenter software (Mashburn,D.N., Lynch,H.E., Ma,X., & Hutson,M.S. Enabling user-guided segmentation and tracking of surface-labeled cells in time-lapse image sets of living tissues. Cytometry A 81, 409-418, 2012).<br><br>Statistical analysis was performed using IBM SPSS Statistics v.22 and Sigmastat version 3.5 (Systat Software). Heat maps were generated using Heatmapper (Babicki,S. et al. Heatmapper: web-enabled heat mapping for all. Nucleic Acids Res. 44, W147-W153 (2016)).

For manuscripts utilizing custom algorithms or software that are central to the research but not yet described in published literature, software must be made available to editors/reviewers. We strongly encourage code deposition in a community repository (e.g. GitHub). See the Nature Research guidelines for submitting code & software for further information.

Data

Policy information about availability of data

All manuscripts must include a data availability statement. This statement should provide the following information, where applicable:

- Accession codes, unique identifiers, or web links for publicly available datasets
- A list of figures that have associated raw data
- A description of any restrictions on data availability

Source data for Figures 1-3, 6-9 and Supplementary Figures are provided as a Source Data file. Additional data that support the findings of this study are available from the corresponding author upon reasonable request.

Field-specific reporting

Please select the one below that is the best fit for your research. If you are not sure, read the appropriate sections before making your selection.

☒ Life sciences ☐ Behavioural & social sciences ☐ Ecological, evolutionary & environmental sciences

For a reference copy of the document with all sections, see nature.com/documents/nr-reporting-summary-flat.pdf

Life sciences study design

All studies must disclose on these points even when the disclosure is negative.

Sample size	Power calculation for sample size estimates for detection of abnormal PNP length were based on previous analysis of PNP length in NTD mouse mutants. Sample size for RNA-seq and qRT-PCR were based on previous informative experiments in models with fully penetrant NTDs. The sample sizes selected produced significant results in analysis of Grhl2 and key known targets (from previously published work) with p<0.01 or small and Power>0.8.
Data exclusions	No data was excluded
Replication	Quantitative measures (PNP length) were made on multiple embryos from multilple litters and repeated in independent experiments. RNA-seq data was validated by qRT-PCR on independent samples. Differential gene/protein expression was further verified by in situ hybridisation and immunohistochemistry on independent embryos fron multiple litters.
Randomization	In culture experiments with blebbistatin, embryos were randomly assigned to treatment groups - the experiments and measurements were blind to genotype (which is determined after the completion of the culture).
Blinding	Measurements of PNP length, puncture assay, closure point ablation, embryo culture and analysis of cellular protrusions were all performed blind to embryo genotype. RNA-seq analysis was performed blind to genotype of experimental groups.

Reporting for specific materials, systems and methods

We require information from authors about some types of materials, experimental systems and methods used in many studies. Here, indicate whether each material, system or method listed is relevant to your study. If you are not sure if a list item applies to your research, read the appropriate section before selecting a response.

Materials & experimental systems

n/a	Involved in the study	
☐	☒	Antibodies
☒	☐	Eukaryotic cell lines
☒	☐	Palaeontology
☐	☒	Animals and other organisms
☒	☐	Human research participants
☒	☐	Clinical data

Methods

n/a	Involved in the study	
☒	☐	ChIP-seq
☒	☐	Flow cytometry
☒	☐	MRI-based neuroimaging

Antibodies

Antibodies used	Primary antibodies were: E-cadherin (610181, BD Biosciences; 3195 Cell signalling), EpCAM (ab71916, Abcam), Cldn 4 (sc17664, Santa Cruz), ZO-1 (402200, Invitrogen), Vimentin (5741, Cell Signaling), Beta-catenin (8814, Cell Signaling), N-Cadherin (14215, Cell Signalling), p-MLCII (3671, Cell Signalling), Sox2 (ab92494, Abcam), fibronectin (sc6952, Santa Cruz).
Validation	E-cadherin (765 citations on manufacturer website), EpCAM (42 references), Cldn4 (9 references) - each show increase/decrease staining in accordance with mRNA level.

ZO-1 (173 references on manufacturer website), Vimentin 546 references (Citeab), beta-catenin (111 references), N-cadherin (34 references; positive neuroepithelium/negative in surface ectoderm), pMLCII (244 references), Sox2 (59 references; validated in Sox2 null tissue in conditional null mouse), fibronectin (23 references)

Animals and other organisms

Policy information about studies involving animals; ARRIVE guidelines recommended for reporting animal research

Laboratory animals	Mice, Strains: Grhl2 gene-trap (AC0205); Axial Defects (both strains on BALB/c genetic background). The study involves analysis of embryos. Experimental litters were generated from intercross of heterozygous mice (females were 8 or more weeks of age).
Wild animals	The study did not involve wild animals
Field-collected samples	The study did not involve samples collected from the wild.
Ethics oversight	Animal studies were approved by the UCL Animal Welfare Ethical Review Body and carried out under regulations of the Animals (Scientific Procedures) Act 1986 of the UK Government, and in accordance with the guidance issued by the Medical Research Council, UK in Responsibility in the Use of Animals for Medical Research (July 1993).

Note that full information on the approval of the study protocol must also be provided in the manuscript.
